# Identification of targetable epigenetic vulnerabilities for uveal melanoma

**DOI:** 10.1038/s41419-025-08295-4

**Published:** 2025-12-12

**Authors:** Gulum Yenisehirli, Sebastian Borges, Steffanie S. Braun, Ashley N. Zuniga, Gabriela I. Quintana, Jeffim N. Kutsnetsoff, Renata L. Volonterio, Sara Rodriguez, Emily V. Adis, Sofia Lopez, James J. Dollar, Vasileios Stathias, Claude-Henry Volmar, Efe Karaca, Shaun P. Brothers, Daniel Bilbao, J. William Harbour, Zelia M. Correa, Stefan Kurtenbach

**Affiliations:** 1 Sylvester Comprehensive Cancer Center (SCCC), University of Miami Miller School of Medicine, Miami, FL USA; 2 Bascom Palmer Eye Institute (BPEI), and Interdisciplinary Stem Cell Institute (ISCI), University of Miami Miller School of Medicine, Miami, FL USA; 3 Department of Molecular and Cellular Pharmacology, University of Miami Miller School of Medicine, Miami, FL USA; 4 Center for Therapeutic Innovation, Department of Psychiatry and Behavioral Sciences, University of Miami Miller School of Medicine, Miami, FL USA; 5 Department of Pathology and Laboratory Medicine, University of Miami Miller School of Medicine, Miami, FL USA; 6 Department of Ophthalmology, University of Texas Southwestern Medical Center, Dallas, TX USA; 7 Simmons Comprehensive Cancer Center, University of Texas Southwestern Medical Center, Dallas, TX USA

**Keywords:** Eye cancer, Cancer genomics, Targeted therapies, High-throughput screening

## Abstract

Uveal melanoma (UM) is the most common adult primary intraocular malignancy, with a strong predilection for hepatic metastasis, occurring in approximately 50% of cases. Metastatic UM is highly resistant to therapy and is almost invariably fatal. The strongest genetic driver of UM metastasis is loss of function of the tumor suppressor BRCA-associated protein 1 (BAP1), which leads to widespread epigenetic dysregulation. To identify novel therapeutic strategies, we investigated whether targeting the epigenome of UM could reveal new vulnerabilities. We performed a high-throughput compound screen using a curated epigenetic inhibitor library and identified BET (bromodomain and extra-terminal domain) inhibition as a particularly promising approach. While previous clinical trials with BET inhibitors for UM treatment have failed, we found substantial heterogeneity in the efficacy of different BET inhibitors in UM. Notably, the BET inhibitor mivebresib (ABBV-075) significantly improved survival rates by 50% in a metastatic UM xenograft mouse model and prevented detectable metastases in the bones, spinal cord, and brain. Transcriptomic analysis revealed a strong overlap between BET and histone deacetylase (HDAC) inhibition, an approach currently under clinical evaluation for UM treatment. BET and HDAC inhibitors reversed gene expression signatures associated with high metastatic risk and induced a neuron-like phenotype in UM cells. These findings establish BET inhibition as a potent and previously underappreciated vulnerability for metastatic UM.

## Introduction

Uveal melanoma (UM) is the most prevalent primary intraocular malignancy in adults, with metastases occurring in approximately half of all cases. UM metastases are highly resistant to treatment and almost uniformly lethal [1]. Currently, the only FDA-approved treatment for metastatic UM is tebentafusp-tebn (Kimmtrak, Immunocore Limited), a bispecific gp100 peptide-HLA-directed CD3 T-cell engager. However, this treatment is only efficient in HLA-A*02:01-positive patients and improves life expectancy by six months on average [2]. Despite this significant advancement, additional treatment strategies are urgently needed.

UM has a low mutational burden, with a mutational profile distinct from other melanomas [3]. Mutually exclusive mutations in the Gq signaling pathway, most commonly in *GNAQ* or *GNA11* [4, 5], and less frequently in *PLCB4* [6] and *CYSLTR2* [7], are present in virtually all UMs [8], but also in benign ocular nevi [4, 5, 8, 9]. Therefore, these mutations alone are insufficient for malignant transformation. Additional secondary mutations in either *BAP1* [10], *SF3B1* [11], or *EIF1AX* [12] (‘BSE’ mutations) occur in a mutually exclusive manner and confer high, medium, and low metastatic risk, respectively [13–15]. *BAP1* mutations are among the most significant clinical markers of metastatic risk, typically accompanied by the loss of one copy of chromosome 3, where *BAP1* is located, resulting in the complete loss of BAP1 function [10]. BAP1 is a ubiquitin carboxy-terminal hydrolase and the catalytic subunit of the polycomb repressive deubiquitinase complex (PR-DUB), which opposes PRC1 activity by removing transcriptionally repressive monoubiquitin marks from histone H2A on K119 [16–18]. BAP1 depletion leads to global changes in H2AK119 ubiquitination [19, 20] and failure of the H3K27ac histone mark to accumulate at promoter sites of key lineage commitment genes, highlighting its broader role in epigenetic regulation [19].

Given the significant role of epigenetic dysregulation in UM [21], we conducted a high-throughput screen for epigenetic modulators. We identify several compounds with high efficacy and highlight BET inhibition as a promising treatment angle for UM.

## Results

### Epigenetic compound screening identifies new vulnerabilities in UM

**Fig. 1:**
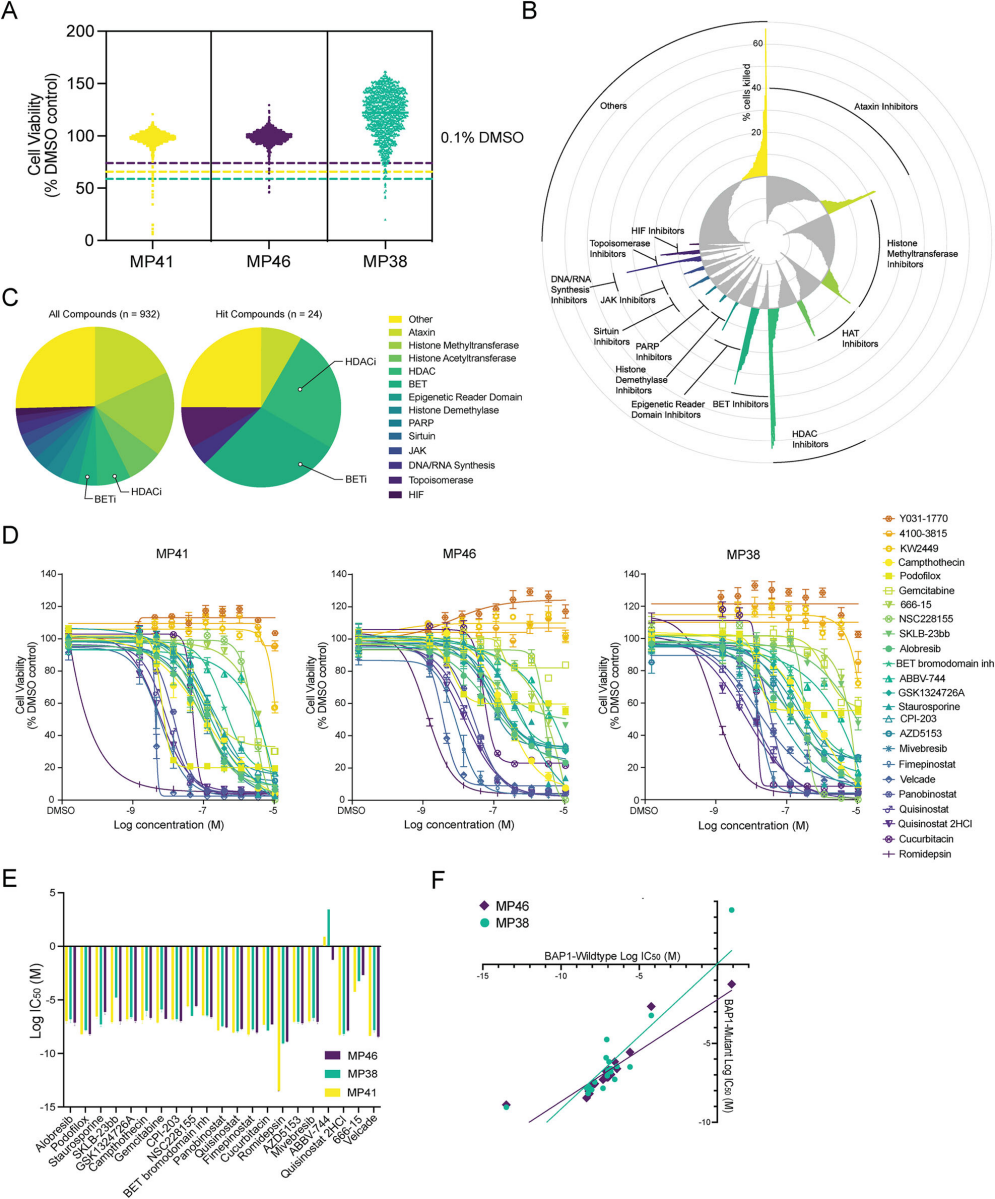
Primary screening for epigenetic compounds in UM cells. **A** Mean viability of the three UM cell lines following 72 h treatment with 932 epigenetic modulators at a concentration of 1 μM (*n* = 2) relative to the negative control (0.1% DMSO treatment). Hit cut-offs (dashed lines) were determined as the mean percentage viability of the negative controls in each cell line minus three standard deviations. Yellow dashed line is the hit cut-off for MP41 cells (65.8% viability), purple dashed line is the hit cut-off for MP46 cells (74.0% viability), and the green dashed line is the hit cut-off for MP38 cells (58.9% viability). For full list of compounds and average UM cell viabilities, see Supplementary Data 1. **B** Radar plot showing the mean difference in percent of cell viability of UM cells caused by 72 h 1 μM treatment with 932 compounds, relative to the DMSO control. Negative values, shown in gray, indicate ineffective compounds leading to greater cell viability than the negative control. The positive values, shown in color, indicate compounds that induced cell death, with higher peaks indicating greater cell death. Compounds are grouped by drug mechanism of action. **C** Pie charts of the molecular activities of all screened compounds (*n* = 932) (left) and the hits identified (*n* = 24) (right). **D** Concentration-response experiments for the 24 hit compounds (10 concentrations, *n* = 4 per concentration per cell line). Center values represent mean viability, error bars represent standard error of mean (SEM). **E** Log IC_50_ (M) values of the top hit compounds for each cell line. Error bars represent 95% confidence interval. **F** Log IC_50_ (M) of *BAP1* mutant cell lines (MP46 and MP38) plotted against the log IC_50_ (M) of the *BAP1* wildtype cell line (MP41) for each drug treatment.

Given the global epigenetic changes elicited by BAP1 loss, we performed a comprehensive epigenetic compound screen on UM cells, using a well-characterized drug library consisting of 932 cell-permeable, small-molecule modulators (TargetMol, L1200, July 2022; Supplementary Data 1). We tested two *BAP1-*mutant UM cell lines (MP38 and MP46) and one *BAP1*-wildtype cell line (MP41) [22]. The primary screen proved to be specific and identified 24 compounds that significantly reduced cell viability in at least one cell line at 1 µM after 72 h of treatment (*n* = 2 per compound) (Fig. 1A). Most drug classes had low efficacy, including histone methyltransferase inhibitors (17% of compounds tested (*n* = 160), 0% of hits), histone acetyltransferase inhibitors (7% of compounds tested (*n* = 68), 0% of hits), and ataxin inhibitors (18% of compounds tested (*n* = 167), 8% of hits (*n* = 2)) (Fig. 1B, C). On the other hand, BET inhibitors (4% of compounds tested, *n* = 35) comprised 29% of the hits (*n* = 7), and HDAC inhibitors (7% of compounds tested, *n* = 64) accounted for 25% of the hits (*n* = 6). Poly ADP-ribose polymerase (PARP) inhibitors (*n* = 28) did not reduce cell viability in these cell lines (Fig. 1B; Supplementary Fig. 1A).

Subsequent concentration-response testing of hit compounds (10 concentrations, *n* = 4) identified 17 compounds with IC_50_ values less than 1 µM (Fig. 1D; Supplementary Table 1). The HDAC inhibitor romidepsin had the highest potency in all UM cell lines (IC_50_ ≈ 4 nM), even greater than that of velcade (IC_50_ ≈ 7.6 nM), a highly cytotoxic proteasome inhibitor [23] used as a positive control in this screen. Eleven of the most promising compounds were HDAC or BET inhibitors, while six compounds targeted other mechanisms. Of the latter, gemcitabine (IC_50_ ≈ 493 nM) and staurosporine (IC_50_ ≈ 336 nM) have previously been shown to induce apoptosis in UM cells [24, 25]. Camptothecin (IC_50_ ≈ 334 nM, topoisomerase I inhibitor [26]), podofilox (IC_50_ ≈ 9.36 nM, microtubule destabilizer [27]), and cucurbitacin B (IC_50_ ≈ 37.9 nM, inhibitor of AKT, HIF1a, and STAT3 [28]), to our knowledge, have not previously been tested for UM. All compounds had similar efficacies in the cell lines tested, despite their genetic differences, namely MP41 being *BAP1*-wildtype and MP38 and MP46 being *BAP1*-mutant (Fig. 1E, F). We tested for synergy between romidepsin and quisinostat with the 12 non-HDAC targeting hit compounds. However, despite these compounds targeting diverse epigenetic pathways, we did not observe significant shifts in IC_50_ values (Supplementary Fig. 1D–G).

### HDAC inhibition in uveal melanoma cells

**Fig. 2:**
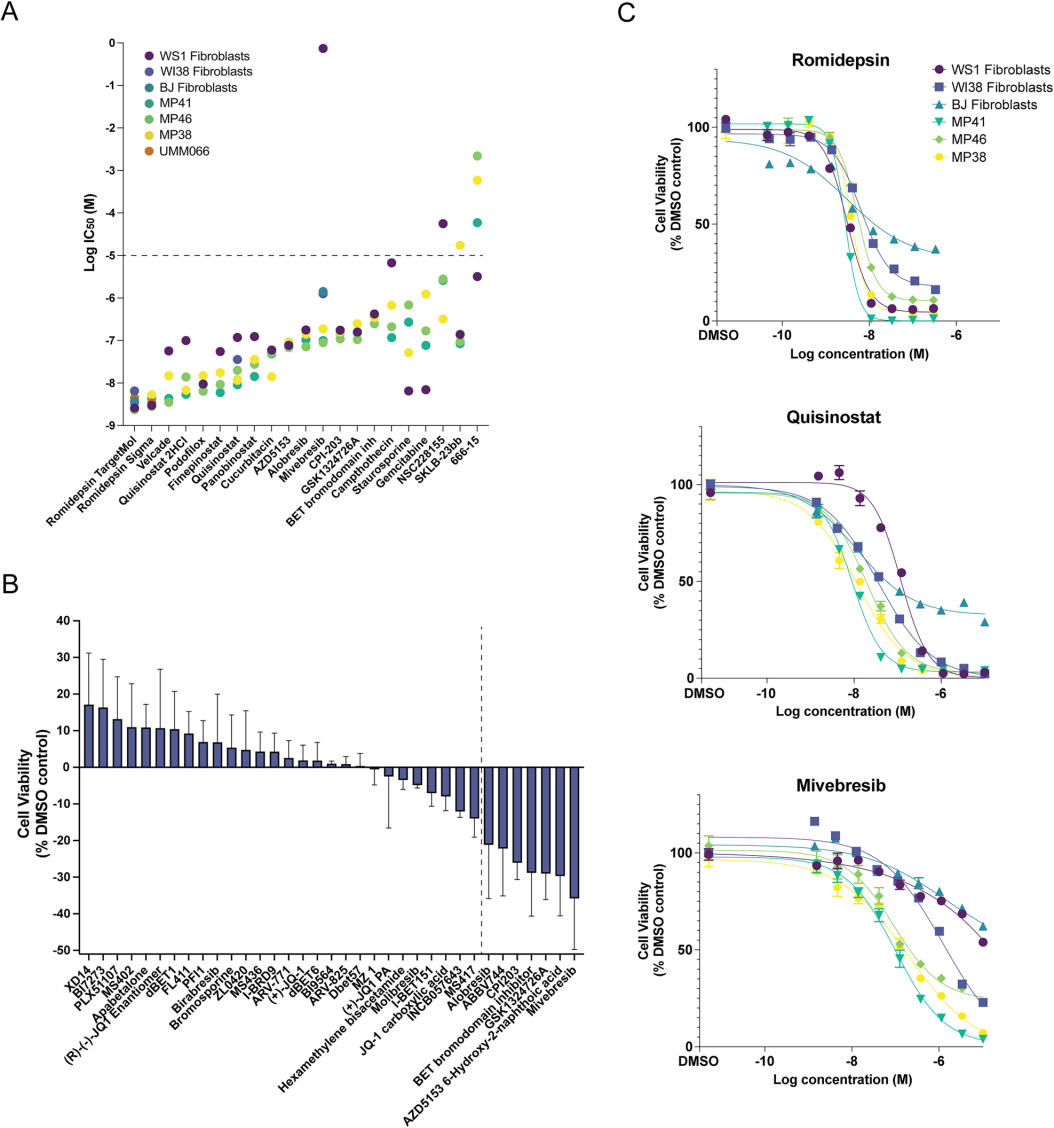
Responses to lead compounds in UM cell lines and normal fibroblasts. **A** Log IC_50_ values of the lead compounds tested in UM cell lines and normal fibroblasts. The dotted line indicates the highest concentration of drug tested (10 μM); hence, for the values above, the IC_50_ was likely not able to be calculated accurately. *n* = 4 replicates for each concentration tested. **B** Mean difference in percent cell viability of three UM cell lines (MP41, MP38, MP46) relative to the DMSO control of all BET inhibitor treatments (72 h, 1 μM) tested in primary screen. Error bars represent SEM. Compounds to the right of the dotted line were identified as hits causing significant cell death in the primary screen. **C** Concentration-response curves of the top candidates (romidepsin, mivebresib, and quisinostat) for UM and fibroblast cell lines. *n* = 4 per concentration tested. Center values represent mean viability, error bars indicate SEM.

HDAC inhibition has been explored in numerous studies, so far with limited clinical success for UM [29–33]. There are 11 human HDAC isoforms with diverse biological functions, and it is unclear which specific HDACs are the most promising to target in UM [34, 35]. Of the 64 HDAC inhibitors tested in the initial screen, only six were identified as hits, highlighting the variable efficacies within this drug class. Romidepsin demonstrated the greatest potency (Fig. 2A, C), suggesting that selective inhibition of class I HDACs may be a vulnerability for UM, as romidepsin specifically inhibits class I HDACs (HDAC1, 2, 3, and 8) [36]. Although no specific inhibitors for HDAC1 and HDAC2 exist to our knowledge, we tested the HDAC3 inhibitor RGFP966 (TargetMol, T1762) and the HDAC8 inhibitor PCI-34051 (TargetMol, T6325) on UM cells and found that neither was potent, alone or in combination (Supplementary Fig. 1B, C). We tested romidepsin from two different sources (TargetMol T6006, Sigma SML1175) and included an additional primary *BAP1*-mutant UM cell line we generated (UMM66) (Fig. 2A). Both romidepsin batches showed similar potency in all cell lines, including UMM66 (IC_50_ = 2.4–5.7 nM). Together, these data highlight romidepsin as the most potent compound in vitro, potentially acting through specific inhibition of class I HDACs, particularly HDAC1 and HDAC2.

### BET inhibition in uveal melanoma cells

To explore non-specific toxicities, we performed viability assays with a non-cancerous WS1 fibroblast cell line (Fig. 2A). Fimepinostat (WS1 IC_50_ ≈ 55 nM, UM IC_50_ ≈ 11 nM) and panobinostat (WS1 IC_50_ ≈ 124 nM, UM IC_50_ ≈ 26 nM) demonstrated 4- to 5-fold lower toxicity to non-transformed cells. Additional drugs with lower cytotoxicity to normal cells included velcade (WS1 IC_50_ ≈ 57 nM, UM IC_50_ ≈ 8 nM), campthothecin (WS1 IC_50_ ≈ 7 µM, UM IC_50_ ≈ 334 nM), and quisinostat (WS1 IC_50_ ≈ 118 nM, UM IC_50_ ≈ 14 nM).

Most of the 35 BET inhibitors tested in the primary screen were not efficient in reducing UM cell viability (Fig. 2B). However, the BET inhibitor mivebresib showed minimal toxicity in normal fibroblasts (WS1 IC_50_ > 10 μM), while being among the most potent BET inhibitors tested (UM IC_50_ ≈ 125 nM) (Fig. 2). These data highlight the significant heterogeneity in the responses of UM cells to different BET inhibitors.

Thus, we selected mivebresib and quisinostat for subsequent testing due to their strong activity in UM cells and lower toxicity to fibroblasts, and included romidepsin due to its high potency and FDA approval for T-cell lymphoma treatment. Romidepsin, quisinostat, and mivebresib were tested on two additional fibroblast cell lines, WI38 and BJ. Consistent with our initial findings, mivebresib exhibited low cytotoxicity in fibroblasts (IC_50_ > 1 μM), while quisinostat demonstrated approximately four-fold selectivity, with an average IC_50_ of 55 nM in fibroblasts compared to 14 nM in UM cells. In contrast, romidepsin showed similar toxicity in both UM and fibroblast cell lines (Fig. 2C). Notably, despite having comparable IC_50_ values to cancer cells, some fibroblasts, particularly BJ cells, exhibited markedly higher resistance to treatment, retaining ~30–40% viability at the highest doses tested, whereas UM cells showed near-complete loss of viability (Fig. 2C).

Although treatment of the primary UM tumors has a high rate of success, approximately half of all patients develop fatal metastases, primarily in the liver. Thus, we tested our lead compounds in a metastatic UM mouse model. We evaluated multiple UM cell lines and found that MP41 cells metastasize predominantly to the liver when injected into the tail vein. MP41 is a *BAP1*-wildtype cell line derived from an aggressive UM case that had spread to multiple organs and has features of *BAP1*-mutant UM, including monosomy 3 [37]. We deemed this model as most suitable to explore the inhibition of metastatic growth in the liver, as we did not find significant differences between MP41 and the *BAP1*-mutant cell lines MP46 and MP38 regarding drug sensitivity.

**Fig. 3:**
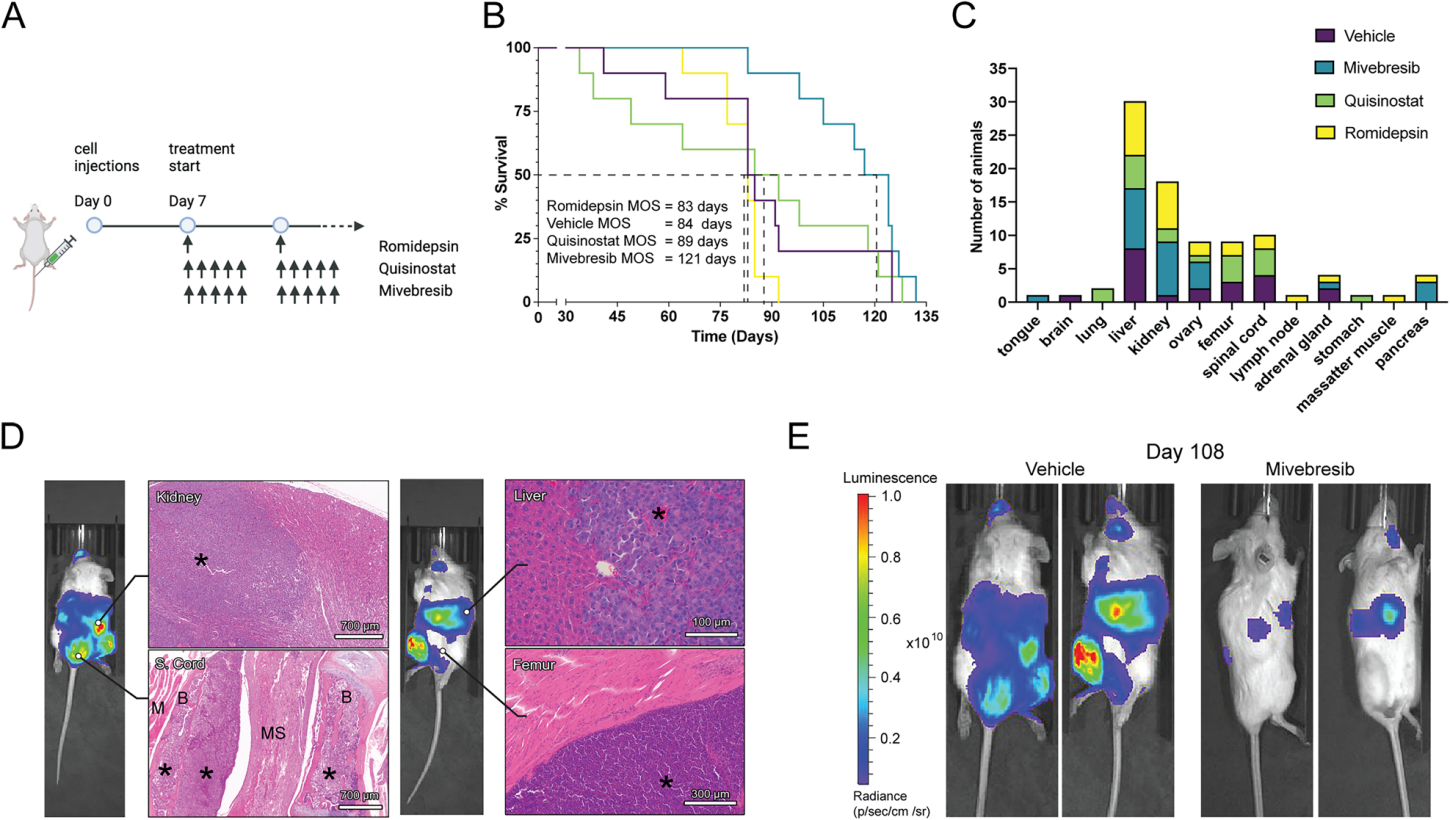
BET inhibition reduces metastatic UM growth in vivo. **A** Experimental outline and timeline of treatments in the metastatic UM mouse model. **B** Percent survival of mice in each treatment group (*n* = 10 per group) over the course of 135 days. Median overall survival (MOS) in days of each treatment group is listed. **C** Bar graph depicting the number of mice in each treatment group with metastatic foci detected in different organs. **D** Representative histopathological images of kidney, spinal cord (S. cord), liver, and femur metastases from the vehicle-treated group. (* = tumor cells; M = muscle; B = bone; MS = medulla spinalis). **E** Representative IVIS images of mice in the vehicle and mivebresib treatment groups on day 108. Luminescence/radiance in p/sec/cm^2^/sr.

Preliminary toxicity assays were conducted to determine optimal drug doses. Drug treatments were initiated seven days after the injection of luciferase-labelled MP41 cells (Fig. 3A). Quisinostat and romidepsin treatments did not significantly improve survival rate (*p* > 0.10), with median survival rates between 83 and 89 days after tumor cell injection (Fig. 3B). Mivebresib treatment improved median survival by nearly 50%, from 84 to 121 days (*p* = 0.01) (Fig. 3B, E). Ex vivo IVIS imaging revealed that mivebresib prevented metastases to the femur and spinal cord, which were detected in all other experimental groups at humane experimental endpoint (Fig. 3C, D).

To test whether long-term in vivo treatments led to the development of drug-resistant metastases, we extracted UM cells from mice liver metastases from all treatment groups and performed concentration-response testing with romidepsin, quisinostat, and mivebresib. No significant resistance was detected in any of the groups relative to cells extracted from mice in the vehicle treatment group (Supplementary Fig. 2).

### Transcriptomic changes associated with HDAC and BET inhibition

**Fig. 4:**
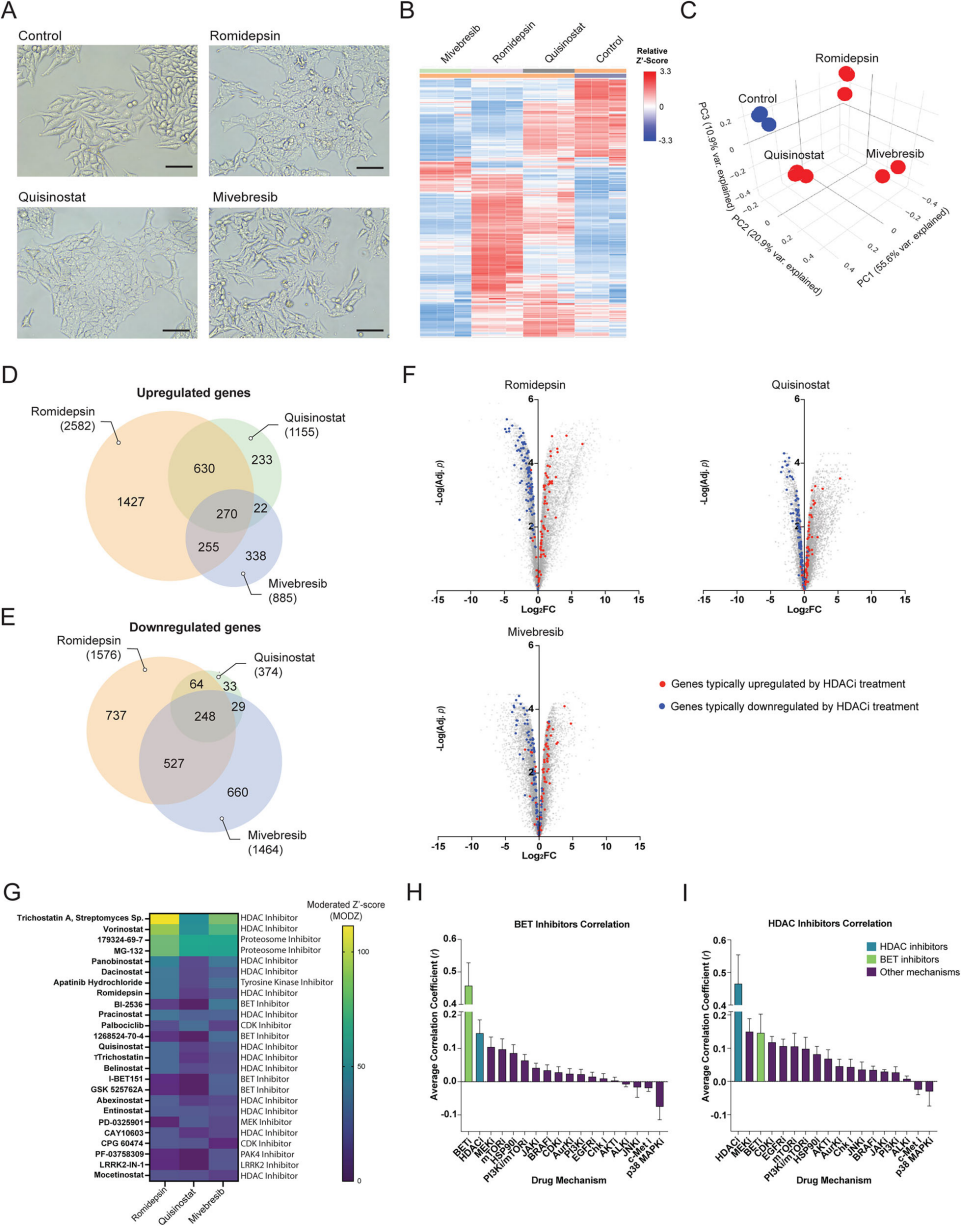
Gene expression changes following BET and HDAC inhibition. **A** Images of MP41 cells treated with each compound for 24 hours. Scale bar = 100 µm. **B** Heatmap clustering of relative Z’-scores for differences in gene expression per treatment group (*n* = 3 per condition). **C** PCA analysis of replicates for each treatment in MP41 cells. **D** Venn diagram depicting overlaps between the treatment groups of significantly upregulated (Adj. *p* < 0.05, log_2_ FC > 1.5) and **E** downregulated genes in MP41 cells (Adj. *p* < 0.05, log_2_ FC < −1.5). **F** Volcano plot of changes in gene expression in MP41 cells relative to the control for each treatment group. Blue and red dots are the 180 genes found to be consistently dysregulated as a result of eight HDAC inhibitor treatments in iLINCS. Red dots are genes that were consistently upregulated by HDAC inhibitor treatments (*n* = 77), while blue dots are genes that were consistently downregulated (*n* = 103). For the HDAC inhibitors considered and transcriptional consensus signatures from which this list was derived, see Supplementary Data 2. For list of genes selected and their direction of change, see Supplementary Data 3. **G** Heatmap of moderated Z’-score (MODZ) of compounds inducing similar gene expression signatures to MP41 cells treated with romidepsin, quisinostat, and mivebresib using iLINCS connected perturbation analysis. Higher MODZ indicates greater similarity. **H** Bar graph of the mean correlation coefficient (*r*) of drugs with each mechanism to BET inhibitors. Error bars represent SEM. **I** Bar graph of the mean correlation coefficient of drugs with each mechanism to HDAC inhibitors. Error bars represent SEM. For full correlation matrix, see Supplementary Data 4.

To elucidate the mechanistic differences of HDAC and BET inhibition in UM, we performed bulk RNA sequencing on UM cell lines treated with romidepsin, quisinostat, and mivebresib. A 24-hour treatment time point was selected to capture early transcriptional responses. Drug concentrations were selected based on preliminary toxicity assays to ensure minimal cell death while eliciting phenotypic effects (Supplementary Fig. 3). Romidepsin, quisinostat, and mivebresib each induced unique morphological changes in MP41 cells, with both HDAC inhibitors causing a flattened morphology, whereas mivebresib-treated cells displayed mixed morphologies including flat and spindle-shaped cells (Fig. 4A). Similar changes were observed in all cell lines, with unique gene expression changes for each compound and a clear separation by principle component analysis (PCA) (Fig. 4B, C; Supplementary Figs. 4B, C and 5B, C). Both HDAC inhibitors resulted in an overall increase in gene expression (Fig. 4D, E; Supplementary Figs. 4D, E and 5D, E), consistent with HDAC inhibitors leading to increased histone acetylation and chromatin accessibility [38]. Mivebresib treatment, on the other hand, resulted in more downregulated than upregulated genes in all cell lines (Fig. 4D, E; Supplementary Figs. 4D, E and 5D, E), in concordance with BET inhibitors preventing the binding of bromodomain (BRD) proteins to acetylated histones, which typically initiate transcription by recruiting transcriptional machinery to acetylated sites [39, 40].

Despite their different mechanisms, we found a significant overlap in gene expression changes elicited by HDAC and BET inhibitors (Fig. 4D, E; Supplementary Figs. 4D, E and 5D, E). To further investigate this finding, we compiled a list of genes consistently up- and down-regulated by HDAC inhibitors across various cancers using the Library of Integrated Network-based Cellular Signature (iLINCS) [41] database, and found that most of these genes were not only up- and down-regulated by HDAC inhibitor treatments in UM cells, but also following BET inhibition with mivebresib (Fig. 4F; Supplementary Fig. 4E, F). We performed an iLINCS connected perturbations analysis, which included gene signatures from various cancer and cell models, and found that mivebresib treatment of UM cells causes a gene expression shift that is most similar to HDAC inhibitors (Fig. 4G; Supplementary Fig. 4G, I). Similarly, correlation analysis of Transcriptional Consensus Signatures (TCS) across compound classes (Supplementary Data 4) revealed BET inhibition to be most similar to HDAC inhibition (*r* = 0.1458) (Fig. 4H). HDAC inhibition was also most similar to MEK (*r* = 0.1494) and BET inhibition (Fig. 4I).

Together, these data show that while BET inhibition may be less toxic and more efficient at reducing the growth of metastatic UM, the gene expression changes elicited by BET and HDAC inhibitors have significant overlap.

### HDAC and BET inhibition reverse transcriptomic signatures associated with high metastatic risk

**Fig. 5:**
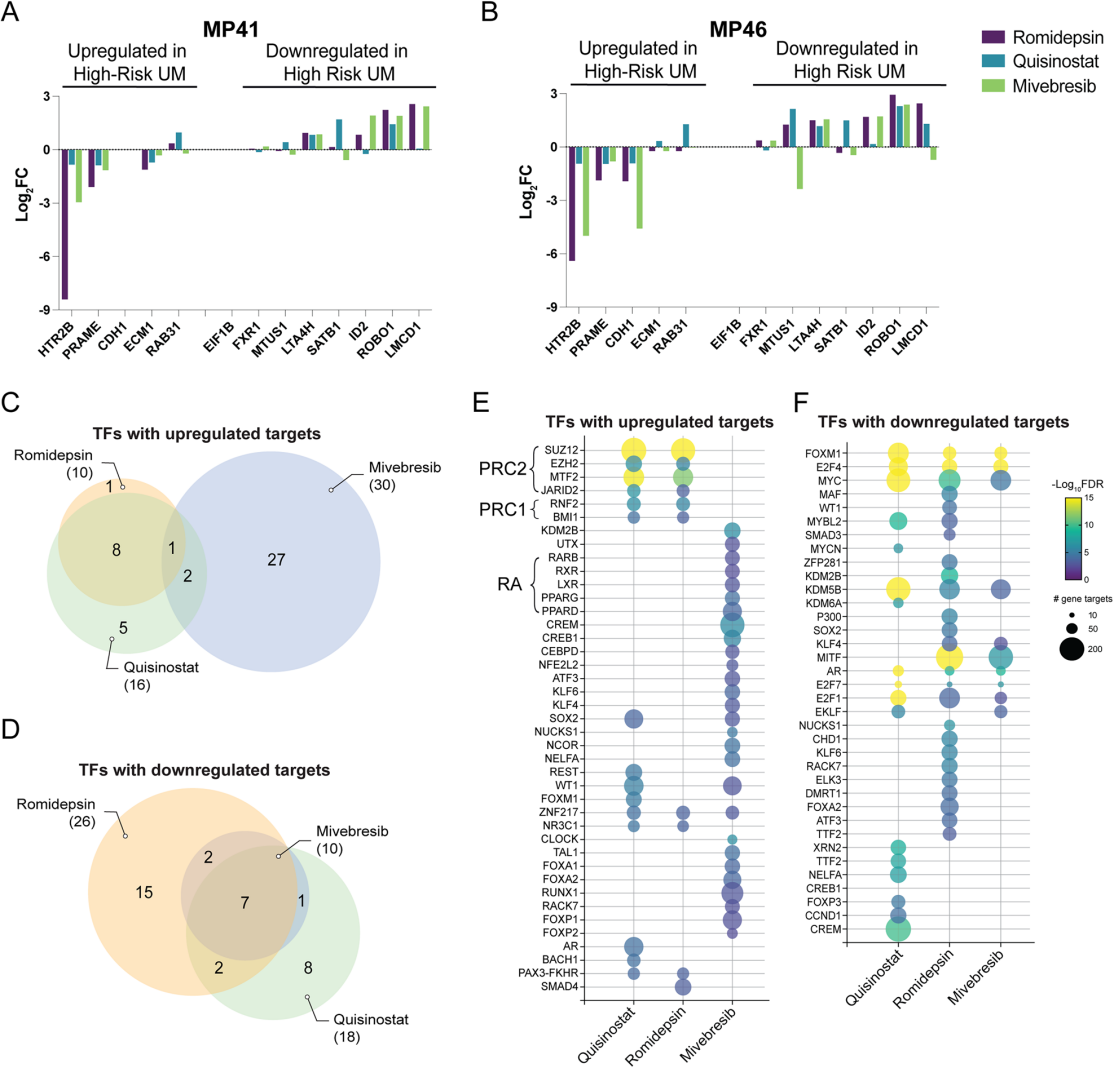
BET and HDAC inhibition reverse high-risk UM signatures through unique mechanisms. **A** Changes in gene expression (log_2_ FC) of genes associated with high-risk UM in drug-treated MP41 and **B** MP46 cells. **C** Venn diagram showing overlaps in predicted transcription factors with upregulated and **D** downregulated gene targets, inferred by gene expression changes induced by each treatment in MP41 cells. **E** Bubble plot of the top predicted transcription factors with upregulated and **F** downregulated gene targets for each treatment in MP41 cells. Color scheme indicates -log_10_ FDR of each predicted transcription factor, and bubble size is determined by the number of corresponding gene targets.

Clinically, UM can be accurately stratified into metastatic risk groups, namely class 1 (low-risk) and class 2 (high-risk), using a gene expression panel of 12 genes [42–44]. An additional biomarker of high metastatic risk for both class 1 and class 2 UM is the expression of *PRAME* [45–47], which is expressed in MP41 and MP46, but not MP38. We found that treatment of UM cells with HDAC and BET inhibitors reversed the high-risk gene expression signature, with high-risk biomarkers such as *HTR2B* and *PRAME* being downregulated (Fig. 5A, B; Supplementary Fig. 5G). Accordingly, genes with low expression in class 2 tumors, such as *ROBO1* and *LMCD1*, were upregulated following treatments (Fig. 5A, B; Supplementary Fig. 5G).

**Fig. 6:**
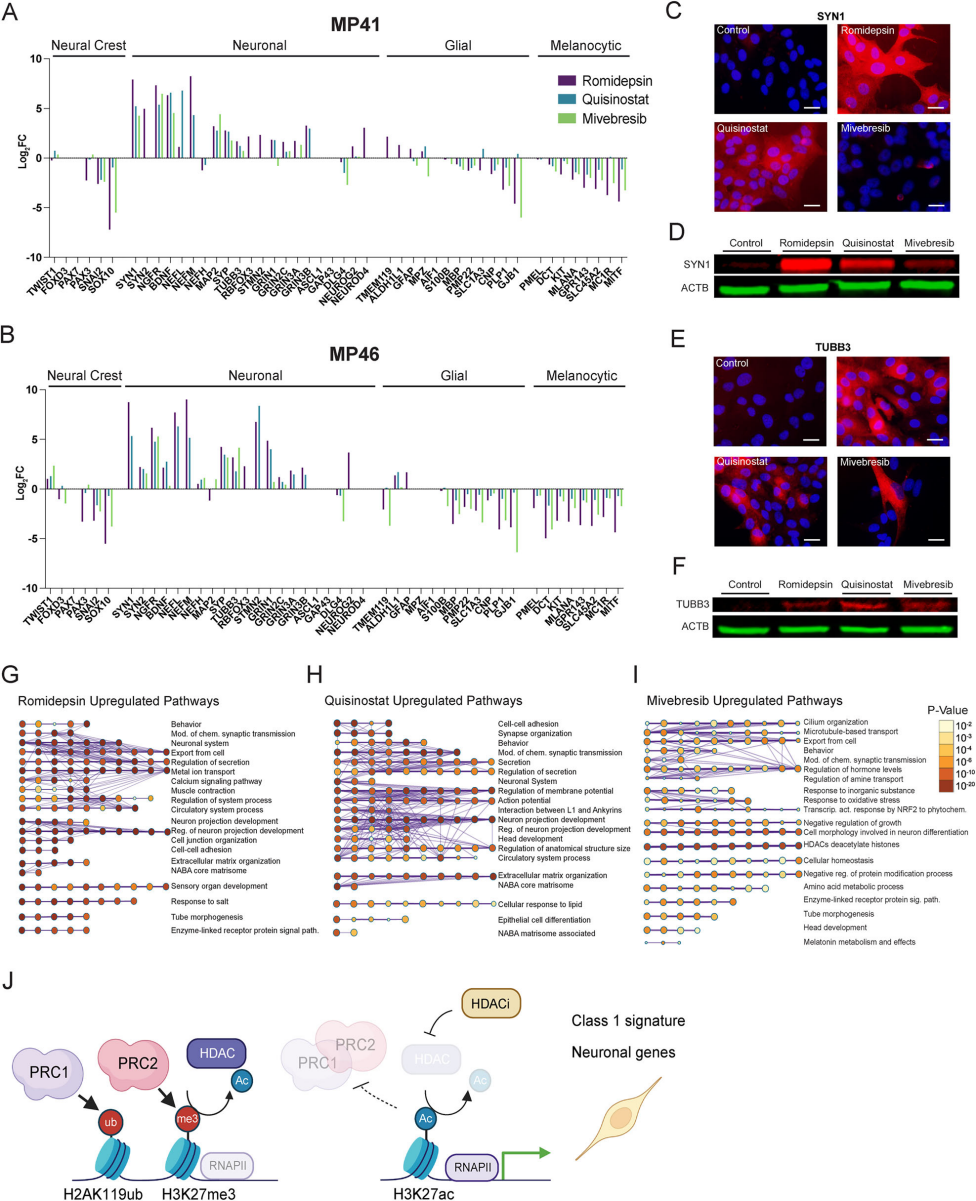
BET and HDAC inhibition induce a neuronal phenotype in UM cells. **A** Changes in the expression (log_2_ FC) of genes associated with some neural-crest-derived cell identities in drug-treated MP41 and **B** MP46 cells. **C** Immunofluorescence images of 24-hour drug-treated MP41 cells. Red fluorescence is Synapsin 1 (SYN1), and blue is DAPI. Scale bar = 25 μm. **D** Immunoblot of SYN1 in 24-hour drug-treated cells with a *β*-Actin (ACTB, green) control. **E** Immunofluorescence images of 24-hour drug-treated MP41 cells. Red fluorescence is *β-*Tubulin III (TUBB3), and blue is DAPI. Scale bar = 25 μm. **F** Immunoblot of TUBB3 in 24-hour drug-treated cells with an ACTB (green) control. **G**–**I** Gene interaction networks of upregulated pathways in MP41 cells predicted from significantly upregulated genes (Adj. *p* < 0.05, log_2_ FC > 1.5) in each treatment group show enrichment for neuronal pathways. **J** Schematic representation of HDAC inhibition impairing PRC activity, leading to elevated expression of PRC target genes, including neuronal genes and those associated with a class 1 phenotype.

ChIP Enrichment Analysis (ChEA) [48] showed that the most prominent increase in gene expression following HDAC treatments were targets of the polycomb repressive complex (PRC) 1 (RNF2, BMI1) and PRC2 (SUZ12, EZH2, and cofactors MTF2, JARID2), indicating a loss of PRC activity (Figs. 5E and 6J; Supplementary Fig. 4J, L). In MP41 cells, the top differentially regulated transcription factor across all treatments was FOXM1, a factor associated with a more aggressive UM phenotype [49], whose target genes were significantly downregulated by all treatments (Fig. 5F). In MP46 cells, on the other hand, the most significant transcription factor whose targets were downregulated in all treatment groups was MITF, indicating decreased melanocytic cell identity (Supplementary Fig. 4K). Other transcription factors whose targets were commonly downregulated by HDAC and BET inhibitor treatment included oncogenic transcription factor MYC and E2F family members E2F1, E2F4, and E2F7 (Fig. 5F). We found a group of unique transcription factors whose activities were upregulated by mivebresib treatment in the PRAME-expressing cell lines MP41 and MP46 (Fig. 5C; Supplementary Fig. 4H). These factors include retinoic acid receptors RXR and RARβ and their binding partners LXR, PPARγ, and PPARδ (Fig. 5E), which regulate pathways involved in neuronal differentiation [50–52].

### BET and HDAC inhibition induce neuronal phenotype in UM cells

We observed that several genes associated with a neuronal cell identity, including *NEFM* (Neuronal Filament Medium), *SYN1* (Synapsin 1), and *NGFR* (Nerve Growth Factor Receptor), were upregulated in UM cells following HDAC or BET inhibitor treatment, more markedly in PRAME-positive cells (Fig. 6A, B). Neural crest and melanocytic identity genes, including *SOX10*, *MLANA*, and *MITF*, were highly downregulated (Fig. 6A, B; Supplementary Fig. 5H). Upregulation of neuronal genes in drug-treated MP41 cells was verified by immunoblotting and immunofluorescence staining of neuronal markers *β*-tubulin III (TUBB3) and Synapsin 1 (SYN1) (Fig. 6C–F). Pathway analysis revealed the upregulation of several neuronal pathways following treatments, including synaptic transmission, neuronal projection, action potential, and neuronal differentiation (Fig. 6G–I; Supplementary Fig. 6D–F, J–M). All drug treatments induced downregulation of pathways involving DNA replication, cell growth, and proliferation (Supplementary Fig. 6).

Together, these data indicate that HDAC and BET inhibition induce a phenotypic identity switch, pushing cells towards a lower metastatic risk gene expression signature and neuronal cell identity (Fig. 6J).

## Discussion

Treatment options for metastatic UM are limited, with the only FDA-approved drug prolonging overall survival by only six months on average for a subset of patients. Here, we utilized an epigenetic compound screen to identify new vulnerabilities to target UM, as most metastatic UM tumors harbor mutations in the chromatin modifier *BAP1*, leading to global epigenomic changes. HDAC and BET inhibitors were the most efficacious compound classes in vitro. We previously showed that PARP inhibition can reduce the metastatic spread in a mouse model of UM [45]. However, our in vitro experiments did not identify PARP inhibitors as a potent drug class (Fig. 1B, C; Supplementary Fig. 1A), indicating that PARP inhibition acts through mechanisms other than reducing UM cell viability.

HDAC inhibitors are widely considered for the treatment of UM [30, 33] with limited clinical success so far. The class I HDAC inhibitor romidepsin was the most potent compound in our screen in vitro (IC_50_ ≈ 4 nM), but it did not improve the survival rate in our metastatic mouse model (Figs. 2A, C and 3B). Romidepsin is FDA-approved for cutaneous T-cell lymphoma treatment [36] and is potent against various other cancer types in vitro [53–55]. In vivo experiments with romidepsin have been challenging, which may be attributed to its short half-life and potential long-term toxicities [56, 57]. However, its high potency in UM cells highlights class I HDAC inhibition specifically as a potential vulnerability in UM and may warrant further studies with different treatment paradigms and delivery systems to identify an applicable therapeutic window.

BET inhibition, on the other hand, has been less explored for UM treatment. While JQ-1 has demonstrated efficacy in UM cells, it is not tested clinically due to its short half-life, although its analogues may be more promising due to their enhanced pharmacokinetic properties [58–60]. Clinical trials with BET inhibitors PLX51107 (NCT02683395) and PLX2853 (NCT03297424), which included UM patients, both had limited success [60–62]. However, we show that BET inhibitors differ significantly in their efficacy for UM. Our initial panel of compounds included 35 BET inhibitors, most of which did not significantly reduce the viability of UM cells (Fig. 2B). Notably, JQ-1, and several of its analogues (MS417, (R)-(-)-JQ1 enantiomer, birabresib, molibresib, ( + )-JQ1 PA, JQ-1 carboxylic acid) were not efficacious, while two JQ-1 analogues, CPI-203 and BET bromodomain inhibitor (CAS: 1505453-59-7), significantly reduced UM cell viability (Fig. 2B). This demonstrates a high level of diversity in compounds within the same drug class, even among the analogues of the same compound. Further pharmacological characterization is required to elucidate the specific molecular features that drive therapeutic response to UM among BET inhibitors.

We find that the BET inhibitor mivebresib has exceptionally low toxicity towards normal fibroblasts and increases the median overall survival time by 50% in a metastatic UM mouse model (Figs. 2A, C and 3B). Mivebresib additionally prevented the development of detectable spinal cord and femur metastases in our mouse model (Fig. 3C). Bone metastasis occurs in approximately 16% of the patients with metastatic UM, and while spinal cord metastases are rare (1%), brain metastases are more frequent (5%) [63, 64]. Although we did not observe frequent brain metastases in our UM model, the blood-spinal cord barrier (BSCB) is similar to the blood-brain barrier (BBB) in function and morphology, potentially indicating that mivebresib may be able to cross the BSCB/BBB more efficiently than the HDAC inhibitors tested [65, 66]. Mivebresib is an oral, small-molecule pan-BET inhibitor that induces cell death and tumor regression in animal models of malignancies such as myeloid leukemia [67], prostate cancer [68], and small cell lung cancer [69]. In a clinical trial for patients with solid tumors that included 10 UM patients, mivebresib prevented tumor growth and reduced tumor volumes in a subset of UM patients. Although associated with manageable but non-trivial adverse events [70], its activity in this high-risk population, together with our preclinical findings, supports further investigation of BET inhibitors as a potentially feasible therapeutic strategy for metastatic UM.

Despite the high in vitro efficacy of HDAC inhibitors quisinostat and romidepsin, these compounds were less effective in improving survival in vivo (Fig. 3B). To elucidate the mechanisms of action of these compounds, we examined the early gene expression changes they induced in UM cells. While each compound elicited unique transcriptional signatures, we identified a significant overlap in the gene expression changes and enriched pathways induced by HDAC and BET inhibition. We found that HDAC inhibition led to the upregulation of PRC1 and PRC2 target genes, whereas BET inhibition acted through other targets, such as retinoic acid-related pathways (Fig. 5E). While promoting cell death, HDAC and BET inhibition initially cause a phenotypic switch, reversing the clinical class 2 (high metastatic risk) gene expression signature (Fig. 5A, B; Supplementary Fig. 5G). The specific reversal of these key markers shows that both drug classes act by initially pushing tumor cells towards a less aggressive class 1 phenotype, rather than being generally toxic. Previous studies have demonstrated that neural progenitor cells treated with HDAC or BET inhibitors favor a neuronal over glial lineage [71–73]. Similarly, we found that genes associated with glial and melanocytic cells were downregulated, while key neuronal genes and pathways were upregulated (Fig. 6; Supplementary Fig. 5H). Notably, BET inhibition led to stronger neuronal gene induction in PRAME-expressing cells, potentially through the restoration of retinoic acid signaling repressed by PRAME [74]. Given the shared developmental origin of melanocytes and some neuronal cell types from neural crest [75], these data indicate that the stem-like features of UM cells [76] may allow them to be pharmacologically pushed towards a neuronal phenotype.

Our data reveal the distinct mechanisms through which HDAC and BET inhibitors reduce the viability of UM cells, and demonstrate the efficacy of the BET inhibitor mivebresib in a metastatic UM mouse model. These findings position BET inhibition as a compelling therapeutic strategy for metastatic UM, warranting further clinical investigation.

## Materials and methods

### Cell culture

UM (MP41, MP46, and MP38) and fibroblast (WI38, WS1, BJ) cell line stocks were obtained from the American Type Culture Collection (ATCC, Manassas, VA, USA). All cells were cultured at 37°C under normoxic conditions (5.0% CO_2_, 20.0% O_2_). Fetal bovine serum (FBS, Neuromics, Edina, MN, USA) used in cell media was heat-inactivated in a water bath at 57°C for 10 minutes. UM cells (MP41, MP46, MP38) were maintained in Dulbecco’s Modified Eagle Medium (D-MEM)/F-12 medium (Thermo Fisher Scientific, Waltham, MA, USA) with 10% heat-inactivated FBS, 2 mmol/L GlutaMAX (Thermo Fisher Scientific), 1 mmol/L Non-Essential Amino Acid (NEAA) cell culture supplement (Cytiva, Marlborough, MA, USA), 0.5× Insulin-Transferrin-Selenium (ITS) (Corning, Corning, NY, USA), and 1× Pen-Strep (10,000 U/mL) (Thermo Fisher Scientific). Fibroblast cell lines (WS1, WI38, and BJ) were cultured separately in Eagle’s Minimum Essential Medium (E-MEM) (ATCC) with 10% heat-inactivated FBS (Neuromics), 1 mmol/L NEAA cell culture supplement (Cytiva), and 1× Pen-Strep (10,000 U/mL, Thermo Fisher Scientific). All cell lines were used at less than 65 passages. All cell lines were verified using short tandem repeat (STR) analysis and tested for mycoplasma contamination with the MycoStrip kit (rep-mysnc-100, Invivogen, San Diego, CA, USA) prior to use.

### Compound screening

For the primary screening, we tested a 932-compound epigenetic library (TargetMol L1200, July 2022; Supplementary Data 1) consisting of inhibitors and activators of epigenetic-modifying enzymes (writers, erasers, and readers). All stock compounds were dissolved in 100% DMSO and tested in duplicates at a test concentration of 1 μM drug and final DMSO concentration of 0.1%. One thousand cells per well were seeded in 384-well white microtiter plates in a humidified incubator at 37°C with 5% O_2_ and 5% CO_2_ overnight (~ 16 h). The cells were then treated with compounds for 72 h. Wells treated with 0.1% DMSO served as negative controls, and velcade (1 μM bortezomib) treatment served as the positive control. Cell viability was assessed by measuring ATP levels using a luminescence-based assay, CellTiter-Glo (Promega, Madison, WI, USA), on a Perkin Elmer Envision Multilabel Plate Reader. Positive hits were compounds that resulted in cell viability falling below the hit cut-offs, which were determined by calculating the mean viability of wells treated with the negative control (0.1% DMSO) for each cell line and subtracting three standard deviations from this mean. This is a stringent cut-off that ensures that only compounds causing a substantial reduction in viability, beyond expected biological variability, are selected. Assays on each plate were considered valid only when the Z’-factor of the plate was equal to or greater than 0.5 (Z’ ≥ 0.5).

### Concentration-response testing

Cell lines were treated using a 10-point 1:3 dilution series starting at a nominal test concentration of 10 μM for all drugs (*n* = 4, 20,000-fold concentration range). Due to the high potency of romidepsin, a lower starting concentration of 300 nM of romidepsin was used in subsequent concentration-response testing. Cell viability was assessed after 72 h of treatment by measuring ATP levels using a luminescence-based assay (CellTiter-Glo, Promega) on a Perkin Elmer Envision Multilabel Plate Reader, and normalized to the viability of cells treated with 0.1% DMSO, which served as the negative control. Four-parameter curve fitting (non-linear regression, log (inhibitor) vs. response, variable slope) was performed using GraphPad Prism to measure the efficacy (% cell viability) and potency (IC_50_) of each compound. Synergy testing with MP38 cells was conducted by first calculating the EC_20_ using non-linear regression (log[agonist] vs. response – FindECanything) in GraphPad Prism based on the concentration-response curves of romidepsin and quisinostat. Concentration–response assays were then conducted for the 12 non-HDAC targeting hit compounds, both alone and in combination with the EC_20_ concentration of either romidepsin or quisinostat. The impact of co-treatment on drug sensitivity was assessed by comparing shifts in IC_50_ values and changes in maximal efficacy (Supplementary Fig. 1D–G).

### Animal studies

The University of Miami Institutional Animal Care and Use Committee (IACUC) approved all animal procedures (reference number: IACUC 21-073). Female NOD Scid Gamma (NSG) mice were obtained from Jackson Laboratory (Stock No. 002374) and bred in-house for one generation. MP41 cells were transduced with retroviruses expressing RFP-luciferase (pMSCV-IRES-luciferase-RFP), and successful transduction was confirmed using an RFP filter on a Zoe cell imager (Bio-Rad, Hercules, CA, USA). RFP-positive cells were sorted and purified using fluorescence-activated cell sorting (FACS), and 100,000 cells were injected intravenously (tail vein) into 16-week-old female NSG mice (*n* = 10 per group). Sample size based on prior studies using this tumor model and experimental design, demonstrating that 10 mice per group are typically sufficient to detect treatment effects ≥40–50% with 80% power at a 5% significance level. Mice were assigned to treatment groups using a pseudo-randomization approach designed to ensure an even distribution of baseline tumor burden. Group allocation was based on the first in vivo bioluminescence imaging (IVIS Spectrum, Revvity) signal (dorsal view), ensuring comparable tumor burden across groups and minimizing allocation bias.

Treatments began seven days post-injection. Drug doses were selected based on prior toxicity testing: 2 mg/kg of romidepsin (TargetMol, T6006) via weekly intraperitoneal (IP) injection, 5 mg/kg of quisinostat (TargetMol, T6055) five times per week via IP injection, and 2 mg/kg of mivebresib (TargetMol, T3712) five times per week via oral gavage. Tumor metastases were monitored weekly during the experiment with IVIS following intraperitoneal injection of d-luciferin (150 mg/kg, Perkin Elmer, 760504). Mice were sacrificed at the humane endpoint (defined as more than 20% weight loss or significant changes in health status, such as paralysis due to spinal cord metastases). No animals were excluded, as no unrelated health issues or technical complications outlined in pre-established exclusion criteria occurred. At the endpoint, metastases in different organs were quantified ex vivo via IVIS. Survival data were analyzed on GraphPad Prism using the log-rank (Mantel-Cox) test, a non-parametric test used to compare survival distributions. All data collection and downstream analyses were performed in a group-blinded manner.

### Isolation and resistance testing of metastatic cells in mouse livers

Following endpoint, tumor-bearing mouse liver tissue was minced and incubated in collagenase Type IV solution (1× D-MEM (Thermo Fisher Scientific) with 400 U/mL Type IV collagenase powder (Thermo Fisher Scientific) and 0.5 μg/mL Amphotericin B solution (Sigma-Aldrich, St. Louis, MO, USA)) overnight at 4°C. Subsequently, cells were seeded in 10 mm tissue culture plates (VWR) in UM cell media and confirmed to be MP41 cells via detection of RFP fluorescence. Drug resistance testing was performed by concentration-response testing (see above), with cells extracted from liver metastases of the vehicle group serving as the control.

### RNA sequencing

For the 24-hour treatment RNA-seq analysis, concentrations were selected through initial testing for concentrations that elicited morphological changes without excessive cell death in 24 hours. Percent cell viabilities for the chosen concentrations were monitored with the Denovix CellDrop FL cell counter by using AOPI (K30520, Denovix, Wilmington, DE, USA) to stain for live and dead cells after 24 h treatment (Supplementary Fig. 3). After concentration selection, 100,000 cells were seeded per well in six-well tissue culture plates (VWR, Radnor, PA, USA) in triplicate for each MP41 and MP46 treatment group (duplicate for MP38). Following cell attachment, cells were treated with romidepsin (40 nM), quisinostat (40 nM), or mivebresib (1200 nM) at a final concentration of 0.1% DMSO in UM media. Wells treated with 0.1% DMSO served as the control group. Total RNA was extracted after 24 hours of treatment using the Quick-RNA MiniPrep kit (Zymo Research, Irvine, CA, USA), and the samples were sequenced by BGI Innomics (Cambridge, MA, USA). All samples were sequenced with over 18 million paired-end reads (150 bp), and passed quality control. The raw files were analyzed using BioJupies to compare each drug-treated group to the control group. Biojupies utilizes *limma*-powered differential expression analysis, which fits a separate linear model to each gene, analyzing all experimental samples simultaneously instead of pairwise comparisons. *Limma* applies moderated t‑tests to compare conditions for each gene, and corrects *p* values for multiple comparisons to obtain adjusted *p* values [77]. Pathway analysis was performed with Metascape [78] using significantly differentially expressed genes (Adj. *p* < 0.05, | log_2_ FC | > 1.5), and transcription factor prediction analysis was performed using ChIP Enrichment Analysis (ChEA) [48].

### iLINCS analysis

To compare the transcriptomic changes caused by our drugs to other perturbations, we used the Library of Integrated Network-based Cellular Signatures (iLINCS) [41] data portal to identify genes dysregulated by HDAC treatments. Using Transcriptional Consensus Signatures (TCS) of HDAC inhibitor treatments obtained from the iLINCS CMAP-L1000 dataset (Supplementary Data 2), we identified 180 genes that were consistently up- or down-regulated as a result of treatment with eight different HDAC treatments (trichostatin A, vorinostat, panobinostat, dacinostat, romidepsin, belinostat, entinostat, mocetinostat) across analyzed cell lines. Genes were selected if they consistently had a positive or negative TCS score across all HDAC treatments (Supplementary Data 3). We then determined the gene expression shifts of these genes as a result of HDAC and BET inhibitor treatment in our cell lines (Fig. 4E, Supplementary Fig. 4E). We additionally used the connected perturbations analysis function of iLINCS to identify compounds eliciting gene signatures similar to those in our study using lists of significantly differentially expressed genes for each treatment group (Adj. *p* < 0.05, | log_2_ FC | > 1.5) (Fig. 4F, Supplementary Fig. 4F). We also used the correlation matrix of TCS’s elicited by compounds in the iLINCS data base (Supplementary Data 4) to calculate the mean correlation coefficient (*r*) of 7 BET inhibitor treatments (JQ-1, (-)-JQ1, (+)-JQ1, JQ1 + SR1277, I-BET, I-BET151, PFI-1) with 7 HDAC inhibitor treatments (entinostat, mocetinostat, rocilinostat, pracinostat, belinostat, vorinostat, MC1568), along with their correlations with the other major drug classes in this dataset (Fig. 4G, H).

### Immunofluorescence of neuronal markers

20,000 MP41 cells per well were seeded in chamber slides (Lab-Tek II, 155382). After cells attached, they were treated with romidepsin (40 nM), quisinostat (40 nM), mivebresib (1200 nM), or 0.01% DMSO (control). Following 24 h treatment, cells were fixed with a 10-minute 4% paraformaldehyde incubation. Immunocytochemistry was performed as described in the Abcam Immunocytochemistry protocol [79]. Cells in each treatment group were incubated with primary antibody for either Synapsin-1 (SYN1, D12G5) (Cell Signaling Technology, Danvers, MA, USA) or *β*-tubulin III (TUBB3, D71G9, Cell Signaling). Alexa-Fluor secondary antibody (IA-11012, Thermo Fisher Scientific) was used for visualization. DAPI (MBD0015, Sigma-Aldrich) diluted 1:10,000 in PBS was added before visualization. The cells were visualized at ×40 on an Olympus CKX53 fluorescent microscope using the Infinity Analyze program.

### Immunoblotting of neuronal markers

100,000 MP41 cells were seeded per well in 6-well tissue culture plates (VWR). After cell attachment, cells were treated with romidepsin (40 nM), quisinostat (40 nM), or mivebresib (1200 nM) at a final concentration of 0.1% DMSO in UM media. Wells treated with 0.1% DMSO served as the control group. Following 24 h treatment, cells were pelleted and lysed with 50 μL of RIPA buffer containing protease inhibitor (Roche Complete ULTRA Tablet, 5892970001). Samples were sonicated and centrifuged at maximum speed (16,000 **×** g) for 15 minutes at 4°C to pellet cellular debris. The supernatants were transferred to new tubes, and protein was quantified with the Pierce BCA assay (Thermo Fisher Scientific). 50 μg of protein was boiled with Laemli buffer + BME at 1/3 of the protein sample for 5 minutes at 95°C. Protein samples were separated on precast polyacrylamide gel (4–15%) (Bio-Rad, 5678084) and transferred to nitrocellulose membrane via Trans-Blot Turbo System (Bio-Rad, 170–4159). Membrane was blocked with 5% bovine serum albumin (BSA) in 0.1% Tween-20 in TBS (TBS-T) for 1 h at room temperature (RT), followed by incubation with primary antibodies for Synapsin-1 (SYN1) (Cell Signaling, D12G5), *β*-tubulin III (TUBB3) (Cell Signaling, D71G9), and *β*-actin (ACTB) (sc-47778, Santa Cruz Biotechnology, Dallas, Texas, USA) diluted in 5% BSA in TBS-T overnight at 4°C. Membranes were washed with TBS-T three times and once with TBS, then incubated in IRDye secondary antibodies (LI-COR, Lincoln, NE, USA, 926-32210, 926-68073) diluted in 5% BSA in TBS-T for 1 h at RT. The membranes were washed with TBS-T three times and once with TBS, then visualized on an Odyssey CLx LI-COR imager.

## Supplementary information

READ ME

Supplementary Table 1

Supplementary Figure 1

Supplementary Figure 2

Supplementary Figure 3

Supplementary Figure 4

Supplementary Figure 5

Supplementary Figure 6

OriginalData

Supplementary Data 1

Supplementary Data 2

Supplementary Data 3

Supplementary Data 4

## Data Availability

Raw and processed RNA sequencing data are available on the Gene Expression Omnibus (GEO) data repository under accession numbers GSE294950 (https://www.ncbi.nlm.nih.gov/geo/query/acc.cgi?acc=GSE294950) for MP41 and MP46 and GSE300710 for MP38 (https://www.ncbi.nlm.nih.gov/geo/query/acc.cgi?acc=GSE300710).

## References

[1] Carvajal RD, Sacco JJ, Jager MJ, Eschelman DJ, Olofsson Bagge R, Harbour JW, et al. Advances in the clinical management of uveal melanoma. Nat Rev Clin Oncol. 2023;20:99–115. 36600005 10.1038/s41571-022-00714-1

[2] Nathan P, Hassel JC, Rutkowski P, Baurain J-F, Butler MO, Schlaak M, et al. Overall survival benefit with tebentafusp in metastatic uveal melanoma. N Engl J Med. 2021;385:1196–206. 34551229 10.1056/NEJMoa2103485

[3] Decatur CL, Ong E, Garg N, Anbunathan H, Bowcock AM, Field MG, et al. Driver mutations in uveal melanoma: associations with gene expression profile and patient outcomes. JAMA Ophthalmol. 2016;134:728–33. 27123562 10.1001/jamaophthalmol.2016.0903 PMC4966162

[4] Van Raamsdonk CD, Bezrookove V, Green G, Bauer J, Gaugler L, O’Brien JM, et al. Frequent somatic mutations of GNAQ in uveal melanoma and blue naevi. Nature. 2009;457:599–602. 19078957 10.1038/nature07586 PMC2696133

[5] Van Raamsdonk CD, Griewank KG, Crosby MB, Garrido MC, Vemula S, Wiesner T, et al. Mutations in GNA11 in uveal melanoma. N Engl J Med. 2010;363:2191–9. 21083380 10.1056/NEJMoa1000584 PMC3107972

[6] Johansson P, Aoude LG, Wadt K, Glasson WJ, Warrier SK, Hewitt AW, et al. Deep sequencing of uveal melanoma identifies a recurrent mutation in PLCB4. Oncotarget. 2016;7:4624. 26683228 10.18632/oncotarget.6614 PMC4826231

[7] Moore AR, Ceraudo E, Sher JJ, Guan Y, Shoushtari AN, Chang MT, et al. Recurrent activating mutations of G-protein-coupled receptor CYSLTR2 in uveal melanoma. Nat Genet. 2016;48:675–80. 27089179 10.1038/ng.3549 PMC5032652

[8] Onken MD, Worley LA, Long MD, Duan S, Council ML, Bowcock AM, et al. Oncogenic mutations in GNAQ occur early in uveal melanoma. Investig Opthalmol Vis Sci. 2008;49:5230. 10.1167/iovs.08-2145 PMC2634606 18719078

[9] Vader M, Madigan M, Versluis M, Suleiman H, Gezgin G, Gruis NA, et al. GNAQ and GNA11 mutations and downstream YAP activation in choroidal nevi. Br J Cancer. 2017;117:884–7. 28809862 10.1038/bjc.2017.259 PMC5590000

[10] Harbour JW, Onken MD, Roberson ED, Duan S, Cao L, Worley LA, et al. Frequent mutation of BAP1 in metastasizing uveal melanomas. SCIENCE. 2010;330:1410–3. 21051595 10.1126/science.1194472 PMC3087380

[11] Harbour JW, Roberson EDO, Anbunathan H, Onken MD, Worley LA, Bowcock AM. Recurrent mutations at codon 625 of the splicing factor SF3B1 in uveal melanoma. Nat Genet. 2013;45:133–5. 23313955 10.1038/ng.2523 PMC3789378

[12] Martin M, Maßhöfer L, Temming P, Rahmann S, Metz C, Bornfeld N, et al. Exome sequencing identifies recurrent somatic mutations in EIF1AX and SF3B1 in uveal melanoma with disomy 3. Nat Genet. 2013;45:933–6. 23793026 10.1038/ng.2674 PMC4307600

[13] Durante MA, Field MG, Sanchez MI, Covington KR, Decatur CL, Dubovy SR, et al. Genomic evolution of uveal melanoma arising in ocular melanocytosis. Mol Case Stud. 2019;5:a004051. 10.1101/mcs.a004051 PMC6672022 31186267

[14] Durante MA, Rodriguez DA, Kurtenbach S, Kuznetsov JN, Sanchez MI, Decatur CL, et al. Single-cell analysis reveals new evolutionary complexity in uveal melanoma. Nat Commun. 2020;11:496. 10.1038/s41467-019-14256-1 PMC6981133 31980621

[15] Field MG, Durante MA, Anbunathan H, Cai LZ, Decatur CL, Bowcock AM, et al. Punctuated evolution of canonical genomic aberrations in uveal melanoma. Nat Commun 2018;9:116. 10.1038/s41467-017-02428-w PMC5760704 29317634

[16] Campagne A, Lee M-K, Zielinski D, Michaud A, Le Corre S, Dingli F, et al. BAP1 complex promotes transcription by opposing PRC1-mediated H2A ubiquitylation. Nat Commun. 2019;10:348. 30664650 10.1038/s41467-018-08255-x PMC6341105

[17] Yu H, Mashtalir N, Daou S, Hammond-Martel I, Ross J, Sui G, et al. The ubiquitin carboxyl hydrolase BAP1 forms a ternary complex with YY1 and HCF-1 and is a critical regulator of gene expression. Mol Cell Biol. 2010;30:5071–85. 20805357 10.1128/MCB.00396-10 PMC2953049

[18] Field MG, Kuznetsov JN, Bussies PL, Cai LZ, Alawa KA, Decatur CL, et al. BAP1 loss is associated with DNA methylomic repatterning in highly aggressive class 2 uveal melanomas. Clin Cancer Res. 2019;25:5663. 31285370 10.1158/1078-0432.CCR-19-0366 PMC6744995

[19] Kuznetsov JN, Aguero TH, Owens DA, Kurtenbach S, Field MG, Durante MA, et al. BAP1 regulates epigenetic switch from pluripotency to differentiation in developmental lineages giving rise to BAP1-mutant cancers. Sci Adv. 2019;5:eaax1738. 31555735 10.1126/sciadv.aax1738 PMC6750916

[20] Bakhoum MF, Francis JH, Agustinus A, Earlie EM, Di Bona M, Abramson DH, et al. Loss of polycomb repressive complex 1 activity and chromosomal instability drive uveal melanoma progression. Nat Commun. 2021;12:5402. 34518527 10.1038/s41467-021-25529-z PMC8438051

[21] Carbone M, Harbour JW, Brugarolas J, Bononi A, Pagano I, Dey A, et al. Biological mechanisms and clinical significance of BAP1 mutations in human cancer. Cancer Discov. 2020;10:1103–20. 32690542 10.1158/2159-8290.CD-19-1220 PMC8006752

[22] Némati F, Sastre-Garau X, Laurent C, Couturier J, Mariani P, Desjardins L, et al. Establishment and characterization of a panel of human uveal melanoma xenografts derived from primary and/or metastatic tumors. Clin Cancer Res. 2010;16:2352–62. 20371695 10.1158/1078-0432.CCR-09-3066

[23] Adams J, Kauffman M. Development of the proteasome inhibitor Velcade™(Bortezomib). Cancer Investig. 2004;22:304–11. 15199612 10.1081/cnv-120030218

[24] Schmittel A, Schmidt-Hieber M, Martus P, Bechrakis N, Schuster R, Siehl J, et al. A randomized phase II trial of gemcitabine plus treosulfan versus treosulfan alone in patients with metastatic uveal melanoma. Ann Oncol. 2006;17:1826–9. 16971664 10.1093/annonc/mdl309

[25] Lapadula D, Farias E, Randolph CE, Purwin TJ, McGrath D, Charpentier TH, et al. Effects of oncogenic Gαq and Gα11 inhibition by FR900359 in uveal melanoma. Mol Cancer Res. 2019;17:963–73. 30567972 10.1158/1541-7786.MCR-18-0574 PMC6445713

[26] Liu LF, Desai SD, LI TK, Mao Y, Sun M, SIM SP. Mechanism of action of camptothecin. Ann N Y Acad Sci. 2000;922:1–10. 11193884 10.1111/j.1749-6632.2000.tb07020.x

[27] Gardner TJ, Cohen T, Redmann V, Lau Z, Felsenfeld D, Tortorella D. Development of a high-content screen for the identification of inhibitors directed against the early steps of the cytomegalovirus infectious cycle. Antivir Res. 2015;113:49–61. 25446405 10.1016/j.antiviral.2014.10.011 PMC4324837

[28] Garg S, Kaul SC, Wadhwa R. Cucurbitacin B and cancer intervention: Chemistry, biology and mechanisms. Int J Oncol. 2018;52:19–37. 29138804 10.3892/ijo.2017.4203

[29] Landreville S, Agapova OA, Matatall KA, Kneass ZT, Onken MD, Lee RS, et al. Histone deacetylase inhibitors induce growth arrest and differentiation in uveal melanoma. Clin cancer Res. 2012;18:408. 22038994 10.1158/1078-0432.CCR-11-0946 PMC3261307

[30] Kuznetsoff JN, Owens DA, Lopez A, Rodriguez DA, Chee NT, Kurtenbach S, et al. Dual screen for efficacy and toxicity identifies HDAC inhibitor with distinctive activity spectrum for BAP1-mutant uveal melanoma. Mol cancer Res. 2021;19:215. 33077485 10.1158/1541-7786.MCR-20-0434 PMC7864865

[31] Moschos MM, Dettoraki M, Androudi S, Kalogeropoulos D, Lavaris A, Garmpis N, et al. The role of histone deacetylase inhibitors in uveal melanoma: current evidence. Anticancer Res. 2018;38:3817–24. 29970501 10.21873/anticanres.12665

[32] Wang Y, Liu M, Jin Y, Jiang S, Pan J. In vitro and in vivo anti-uveal melanoma activity of JSL-1, a novel HDAC inhibitor. Cancer Lett. 2017;400:47–60. 28455241 10.1016/j.canlet.2017.04.028

[33] Dai W, Zhou J, Jin B, Pan J. Class III-specific HDAC inhibitor Tenovin-6 induces apoptosis, suppresses migration and eliminates cancer stem cells in uveal melanoma. Sci Rep. 2016;6:22622. 26940009 10.1038/srep22622 PMC4778058

[34] Nicolas E, Yamada T, Cam HP, FitzGerald PC, Kobayashi R, Grewal SI. Distinct roles of HDAC complexes in promoter silencing, antisense suppression and DNA damage protection. Nat Struct Mol Biol. 2007;14:372–80. 17450151 10.1038/nsmb1239

[35] Witt O, Deubzer HE, Milde T, Oehme I. HDAC family: what are the cancer relevant targets?. Cancer Lett. 2009;277:8–21. 18824292 10.1016/j.canlet.2008.08.016

[36] VanderMolen KM, McCulloch W, Pearce CJ, Oberlies NH. Romidepsin (Istodax, NSC 630176, FR901228, FK228, depsipeptide): a natural product recently approved for cutaneous T-cell lymphoma. J Antibiot. 2011;64:525–31. 10.1038/ja.2011.35 PMC3163831 21587264

[37] Gentien D, Saberi-Ansari E, Servant N, Jolly A, de la Grange P, Némati F, et al. Multi-omics comparison of malignant and normal uveal melanocytes reveals molecular features of uveal melanoma. Cell Rep. 2023;42:113132. 10.1016/j.celrep.2023.113132 PMC10598242 37708024

[38] Slaughter MJ, Shanle EK, Khan A, Chua KF, Hong T, Boxer LD, et al. HDAC inhibition results in widespread alteration of the histone acetylation landscape and BRD4 targeting to gene bodies. Cell Rep. 2021;34:108638. 10.1016/j.celrep.2020.108638 PMC7886050 33472068

[39] Dhalluin C, Carlson JE, Zeng L, He C, Aggarwal AK, Zhou M-M, et al. Structure and ligand of a histone acetyltransferase bromodomain. Nature. 1999;399:491–6. 10365964 10.1038/20974

[40] Yang Z, Yik JH, Chen R, He N, Jang MK, Ozato K, et al. Recruitment of P-TEFb for stimulation of transcriptional elongation by the bromodomain protein Brd4. Mol Cell. 2005;19:535–45. 16109377 10.1016/j.molcel.2005.06.029

[41] Pilarczyk M, Fazel-Najafabadi M, Kouril M, Shamsaei B, Vasiliauskas J, Niu W, et al. Connecting omics signatures and revealing biological mechanisms with iLINCS. Nat Commun. 2022;13:4678. 35945222 10.1038/s41467-022-32205-3 PMC9362980

[42] Onken MD, Worley LA, Ehlers JP, Harbour JW. Gene expression profiling in uveal melanoma reveals two molecular classes and predicts metastatic death. Cancer Res. 2004;64:7205–9. 15492234 10.1158/0008-5472.CAN-04-1750 PMC5407684

[43] Harbour JW. A prognostic test to predict the risk of metastasis in uveal melanoma based on a 15-gene expression profile. Methods Mol. Biol. 2014:427–40. 10.1007/978-1-62703-727-3_22 PMC4476294 24258991

[44] Harbour JW, Chen R. The DecisionDx-UM gene expression profile test provides risk stratification and individualized patient care in uveal melanoma. PLoS Curr 2013;5:ecurrents.eogt.af8ba80fc776c8f1ce8f5dc485d4a618. 10.1371/currents.eogt.af8ba80fc776c8f1ce8f5dc485d4a618 PMC3625622 23591547

[45] Kurtenbach S, Sanchez MI, Kuznetsoff J, Rodriguez DA, Weich N, Dollar JJ, et al. PRAME induces genomic instability in uveal melanoma. Oncogene. 2023:1–11. 10.1038/s41388-023-02887-0 PMC10873199 38030788

[46] Field MG, Decatur CL, Kurtenbach S, Gezgin G, Van Der Velden PA, Jager MJ, et al. PRAME as an independent biomarker for metastasis in uveal melanoma. Clin Cancer Res. 2016;22:1234–42. 26933176 10.1158/1078-0432.CCR-15-2071 PMC4780366

[47] Harbour JW, Correa ZM, Schefler AC, Mruthyunjaya P, Materin MA, Aaberg TA, Jr. et al. 15-gene expression profile and PRAME as integrated prognostic test for uveal melanoma: first report of collaborative ocular oncology group study no. 2 (COOG2.1). J Clin Oncol. 2024:JCO2400447. 10.1200/JCO.24.00447 PMC11421563 39052972

[48] Lachmann A, Xu H, Krishnan J, Berger SI, Mazloom AR, Ma’ayan A. ChEA: transcription factor regulation inferred from integrating genome-wide ChIP-X experiments. Bioinformatics. 2010;26:2438–44. 20709693 10.1093/bioinformatics/btq466 PMC2944209

[49] Bai X, Li S, Luo Y. FOXM1 promote the growth and metastasis of uveal melanoma cells by regulating CDK2 expression. Int Ophthalmol. 2024;44:55. 38342795 10.1007/s10792-024-02943-y PMC10859341

[50] Quintanilla RA, Utreras E, Cabezas-Opazo FA. Role of PPARγ in the differentiation and function of neurons. PPAR Res. 2014;2014:768594. 25246934 10.1155/2014/768594 PMC4160645

[51] Simandi Z, Horvath A, Cuaranta-Monroy I, Sauer S, Deleuze J-F, Nagy L. RXR heterodimers orchestrate transcriptional control of neurogenesis and cell fate specification. Mol Cell Endocrinol. 2018;471:51–62. 28778663 10.1016/j.mce.2017.07.033

[52] Schmidt A, Vogel R, Holloway MK, Rutledge SJ, Friedman O, Yang Z, et al. Transcription control and neuronal differentiation by agents that activate the LXR nuclear receptor family. Mol Cell Endocrinol. 1999;155:51–60. 10580838 10.1016/s0303-7207(99)00115-x

[53] Mayr C, Kiesslich T, Erber S, Bekric D, Dobias H, Beyreis M, et al. HDAC screening identifies the HDAC class I inhibitor romidepsin as a promising epigenetic drug for biliary tract cancer. Cancers. 2021;13:3862. 34359763 10.3390/cancers13153862 PMC8345689

[54] Panicker J, Li Z, McMahon C, Sizer C, Steadman K, Piekarz R, et al. Romidepsin (FK228/depsipeptide) controls growth and induces apoptosis in neuroblastoma tumor cells. Cell cycle. 2010;9:1830–8. 20404560 10.4161/cc.9.9.11543 PMC6659113

[55] Li L-H, Zhang P-R, Cai P-Y, Li Z-C. Histone deacetylase inhibitor, Romidepsin (FK228) inhibits endometrial cancer cell growth through augmentation of p53-p21 pathway. Biomed Pharmacother. 2016;82:161–6. 27470351 10.1016/j.biopha.2016.04.053

[56] Rivers ZT, Oostra DR, Westholder JS, Vercellotti GM. Romidepsin-associated cardiac toxicity and ECG changes: a case report and review of the literature. J Oncol Pharm Pract. 2018;24:56–62. 27708192 10.1177/1078155216673229

[57] Klimek VM, Fircanis S, Maslak P, Guernah I, Baum M, Wu N, et al. Tolerability, pharmacodynamics, and pharmacokinetics studies of depsipeptide (romidepsin) in patients with acute myelogenous leukemia or advanced myelodysplastic syndromes. Clin Cancer Res. 2008;14:826–32. 18245545 10.1158/1078-0432.CCR-07-0318

[58] Liu W, Cui Z, Wan Q, Liu Y, Chen M, Cheng Y, et al. The BET inhibitor JQ1 suppresses tumor survival by ABCB5-mediated autophagy in uveal melanoma. Cell Signal. 2025;125:111483. 39442901 10.1016/j.cellsig.2024.111483

[59] Chen X, Huang R, Zhang Z, Song X, Shen J, Wu Q. Bet bromodomain inhibition potentiates ocular melanoma therapy by inducing cell cycle arrest. Investig Ophthalmol Vis Sci. 2024;65:11. 10.1167/iovs.65.8.11 PMC11232900 38967943

[60] Croce M, Ferrini S, Pfeffer U, Gangemi R. Targeted therapy of uveal melanoma: Recent failures and new perspectives. Cancers. 2019;11:846. 31216772 10.3390/cancers11060846 PMC6628160

[61] Patnaik A, Carvajal RD, Komatsubara KM, Britten CD, Wesolowski R, Michelson G, et al. Phase ib/2a study of PLX51107, a small molecule BET inhibitor, in subjects with advanced hematological malignancies and solid tumors. Am Soc Clin Oncol 2018;36:2550.

[62] Liu XL, Run-Hua Z, Pan JX, Li ZJ, Yu L, Li YL. Emerging therapeutic strategies for metastatic uveal melanoma: targeting driver mutations. Pigment Cell Melanoma Res. 2024;37:411–25. 38411373 10.1111/pcmr.13161

[63] Group TCOMS. Assessment of metastatic disease status at death in 435 patients with large choroidal melanoma in the collaborative ocular melanoma study (COMS): COMS report no. 15. Arch Ophthalmol. 2001;119:670–6. 11346394 10.1001/archopht.119.5.670

[64] Wei AZ, Uriel M, Porcu A, Manos MP, Mercurio AC, Caplan MM, et al. Characterizing metastatic uveal melanoma patients who develop symptomatic brain metastases. Front Oncol. 2022;12:961517. 36212499 10.3389/fonc.2022.961517 PMC9540230

[65] Sullivan JM, Badimon A, Schaefer U, Ayata P, Gray J, Chung C-w, et al. Autism-like syndrome is induced by pharmacological suppression of BET proteins in young mice. J Exp Med. 2015;212:1771–81. 26392221 10.1084/jem.20151271 PMC4612093

[66] Govindarajan V, Shah AH, Di L, Rivas S, Suter RK, Eichberg DG, et al. Systematic review of epigenetic therapies for treatment of IDH-mutant glioma. World Neurosurg. 2022;162:47–56. 35314408 10.1016/j.wneu.2022.03.051 PMC9177782

[67] Albert DH, Goodwin NC, Davies AM, Rowe J, Feuer G, Boyiadzis M, et al. Co-clinical modeling of the activity of the BET inhibitor mivebresib (ABBV-075) in AML. vivo. 2022;36:1615–27. 10.21873/invivo.12872 PMC9301399 35738590

[68] Faivre EJ, Wilcox D, Lin X, Hessler P, Torrent M, He W, et al. Exploitation of castration-resistant prostate cancer transcription factor dependencies by the novel BET inhibitor ABBV-075. Mol Cancer Res. 2017;15:35–44. 27707886 10.1158/1541-7786.MCR-16-0221

[69] Lam LT, Lin X, Faivre EJ, Yang Z, Huang X, Wilcox DM, et al. Vulnerability of small-cell lung cancer to apoptosis induced by the combination of BET bromodomain proteins and BCL2 inhibitors. Mol Cancer Ther. 2017;16:1511–20. 28468776 10.1158/1535-7163.MCT-16-0459

[70] Piha-Paul SA, Sachdev JC, Barve M, LoRusso P, Szmulewitz R, Patel SP, et al. First-in-human study of mivebresib (ABBV-075), an oral pan-inhibitor of bromodomain and extra terminal proteins, in patients with relapsed/refractory solid tumors. Clin Cancer Res. 2019;25:6309–19. 31420359 10.1158/1078-0432.CCR-19-0578

[71] Siebzehnrubl FA, Buslei R, Eyupoglu IY, Seufert S, Hahnen E, Blumcke I. Histone deacetylase inhibitors increase neuronal differentiation in adult forebrain precursor cells. Exp Brain Res. 2007;176:672–8. 17216146 10.1007/s00221-006-0831-x

[72] Hsieh J, Nakashima K, Kuwabara T, Mejia E, Gage FH. Histone deacetylase inhibition-mediated neuronal differentiation of multipotent adult neural progenitor cells. Proc Natl Acad Sci. 2004;101:16659–64. 15537713 10.1073/pnas.0407643101 PMC527137

[73] Li J, Ma J, Meng G, Lin H, Wu S, Wang J, et al. BET bromodomain inhibition promotes neurogenesis while inhibiting gliogenesis in neural progenitor cells. Stem Cell Res. 2016;17:212–21. 27591477 10.1016/j.scr.2016.07.006

[74] Epping MT, Wang L, Edel MJ, Carlée L, Hernandez M, Bernards R. The human tumor antigen PRAME is a dominant repressor of retinoic acid receptor signaling. Cell. 2005;122:835–47. 16179254 10.1016/j.cell.2005.07.003

[75] Le Douarin N, Kalcheim C. The neural crest: Cambridge University Press; 1999.

[76] Matatall KA, Agapova OA, Onken MD, Worley LA, Bowcock AM, Harbour JW. BAP1 deficiency causes loss of melanocytic cell identity in uveal melanoma. BMC Cancer. 2013;13:1–12. 23915344 10.1186/1471-2407-13-371 PMC3846494

[77] Torre D, Lachmann A, Ma’ayan A. BioJupies: automated generation of interactive notebooks for RNA-Seq data analysis in the cloud. Cell Syst. 2018;7:556–61.e3. 30447998 10.1016/j.cels.2018.10.007 PMC6265050

[78] Zhou Y, Zhou B, Pache L, Chang M, Khodabakhshi AH, Tanaseichuk O, et al. Metascape provides a biologist-oriented resource for the analysis of systems-level datasets. Nat Commun. 2019;10:1523. 30944313 10.1038/s41467-019-09234-6 PMC6447622

[79] Abcam. Immunocytochemistry protocol: Abcam; 2022 [Available from: https://www.abcam.com/en-us/technical-resources/protocols/icc-protocol?srsltid=AfmBOoqyt4JzQIg_lE0OOXDD4ho0XaMzPhyNsZIHA97qu47Q0IKgCbGx.

