# Supplementary material for: Identification of targetable epigenetic vulnerabilities for uveal melanoma
Source: Cell Death Dis. 2025 Dec 12;17(1):89. doi: 10.1038/s41419-025-08295-4 (PMC12830624; doi:10.1038/s41419-025-08295-4)
Supplement: Supplementary file 1 — READ ME [file 41419_2025_8295_MOESM1_ESM.pdf]

Supplementary Table 1. Hit compounds screen results. IC_{50} (M) values of the 24 hit compounds identified by the primary screen for each UM cell line and mean IC_{50}, along with the mechanisms of action for each compound.

Supplementary Figure 1. Additional concentration-response testing. (A) Concentration-response curves of MP41 and MP38 cells treated with the PARP inhibitor talazoparib. Center values represent mean, and error bars indicate SEM. n=4 for each concentration. **(B)** Concentration-response curves of MP41 and **(C)** MP38 cells treated with HDAC3 (RGFP966) and HDAC8 (PCI-34051) inhibitors. Center values represent mean, and error bars indicate SEM. n=4 for each concentration. **(D)** Mean difference in percent cell viability at the highest concentration (10 µM) for MP38 cells treated with quisinostat plus EC_{20} of other candidate compounds relative to cell viability when treated with only 10 µM quisinostat. Greater positive values indicate better synergy. **(E)** Mean difference in percent cell viability at the highest concentration (10 µM) for MP38 cells treated with romidepsin plus EC_{20} of other candidate compounds relative to cell viability when treated with only 10 µM romidepsin. Greater positive values indicate better synergy. **(F)** Mean log IC_{50} shift of MP38 cells treated with Quisinostat and the EC_{20} of other candidate compounds relative to cells treated with only quisinostat. Greater positive values indicate better synergy. **(G)** Mean log IC_{50} shift of MP38 cells treated with romidepsin and the EC_{20} of other candidate compounds relative to cells treated with only romidepsin. Greater positive values indicate better synergy.

Supplementary Figure 2. *Ex vivo* testing of acquired drug resistance in tumor cells from vehicle and drug-treated murine livers. (A) Concentration-response curves for quisinostat treatment of MP41 cells extracted from mouse liver tumor samples (vehicle n =3; quisinostat n =1, mivebresib n = 4, romidepsin n = 3). Center values represent mean, and error bars indicate SEM. **(B)** Concentration-response curves for romidepsin treatment of MP41 cells extracted from mouse liver tumor samples (vehicle n =3; quisinostat n =1, mivebresib n = 4, romidepsin n = 3). Center values represent mean, and error bars indicate SEM. **(C)** Concentration-response curve of mivebresib treatment of MP41 cells extracted from mouse liver tumor samples (vehicle n =3; quisinostat n =1, mivebresib n = 4, romidepsin n = 3). Center values represent mean, and error bars indicate SEM.

Supplementary Figure 3. Live/dead percentages of UM cells after treatments for RNA sequencing. (A) Mean percent of live and dead cells under all RNA-seq preparation conditions (24 h treatment with control (0.1% DMSO), 1 200 nM mivebresib, 40 nM quisinostat, or 40 nM romidepsin) for MP41, **(B)** MP38, and **(C)** MP46 cells. Error bars indicate SEM.

Supplementary Figure 4. RNA-seq analysis of MP46 cells treated with BET and HDAC inhibitors. (A) Images of MP46 cells treated with each compound for 24 hours. Scale bar = 100 µm. **(B)** Heatmap clustering of relative Z'-scores for changes in gene expression of MP46 cells per treatment group (n = 3 per condition). **(C)** PCA clustering of replicates for each treatment in MP46 cells. **(D)** Venn diagram depicting overlaps between the treatment groups of significantly upregulated (Adj. $p < 0.05$, $\log_2 FC > 1.5$). and **(E)** downregulated genes in drug-treated MP46 cells (Adj. $p < 0.05$, $\log_2 FC < -1.5$). **(F)** Volcano plot of changes in gene expression relative to the control for each treatment group in MP46 cells. Blue and red dots are 180 genes found to be consistently dysregulated as a result of eight HDAC inhibitor treatments in iLINCS. Red dots are genes that were consistently upregulated by HDAC inhibitor treatments (n = 77), while blue dots are genes that were consistently downregulated (n = 103). For the HDAC inhibitors considered and transcriptional consensus signatures from which this list was derived, see Supplementary Data 2. For list of genes selected and their direction of change, see Supplementary Data 3. **(G)** Heatmap of moderated Z'-score (MODZ) of compounds inducing similar gene expression signatures to romidepsin, quisinostat, and mivebresib treatment in MP46 cells using iLINCS connected perturbation analysis. Higher MODZ indicates greater similarity. **(H)** Venn diagram showing overlaps in predicted transcription factors with upregulated and **(I)** downregulated gene targets, inferred by gene expression changes induced by each treatment in MP46 cells. **(J)** Bubble plot of the top predicted transcription factors with upregulated and **(K)** downregulated gene targets for each treatment in MP46 cells. Color scheme indicates $-\log_{10}$ FDR of each predicted transcription factor, and bubble size is determined by the number of corresponding gene targets.

Supplementary Figure 5. RNA-seq analysis of MP38 cells treated with BET and HDAC inhibitors. (A) Images of MP38 cells treated with each compound for 24 hours. Scale bar = 100 µm. **(B)** Heatmap clustering of relative Z'-scores for changes in gene expression of MP38 cells per treatment group (n = 3 per condition). **(C)** PCA clustering of replicates for each treatment in MP38 cells. **(D)** Venn diagram depicting overlaps between the treatment groups of significantly upregulated (Adj. $p < 0.05$, $\log_2 FC > 1.5$). and **(E)** downregulated genes in drug-treated MP38 cells (Adj. $p < 0.05$, $\log_2 FC < -1.5$). **(F)** Volcano plot of changes in gene expression relative to the control for each treatment group in MP38 cells. Blue and red dots are 180 genes found to be consistently dysregulated as a result of eight HDAC inhibitor treatments in iLINCS. Red dots are genes that were consistently upregulated by HDAC inhibitor treatments (n = 77), while blue dots are genes that were consistently downregulated (n = 103). For the HDAC inhibitors considered and transcriptional consensus signatures from which this list was derived, see Supplementary Data 2. For list of genes selected and their direction of change, see Supplementary Data 3. **(G)** Changes in gene expression ($\log_2$ FC) of genes associated with high-risk UM in drug-treated MP38 cells. **(H)** Changes in the expression ($\log_2$ FC) of genes associated with some neural-crest-derived cell identities in drug-treated MP38 cells. **(I)** Heatmap of moderated Z'-score (MODZ) of compounds inducing similar gene expression signatures to romidepsin, quisinostat, and mivebresib treatment in MP38 cells using iLINCS connected perturbation analysis. Higher MODZ indicates greater similarity. **(J)** Venn diagram showing overlaps in predicted transcription factors

with upregulated and **(K)** downregulated gene targets, inferred by gene expression changes induced by each treatment in MP38 cells. **(L)** Bubble plot of the top predicted transcription factors with upregulated and **(M)** downregulated gene targets for each treatment in MP38 cells. Color scheme indicates $-\log_{10}$ FDR of each predicted transcription factor, and bubble size is determined by the number of corresponding gene targets.

Supplementary Figure 6. Pathway changes induced by BET and HDAC inhibitors in UM. (A-C) Downregulated pathways in drug-treated MP41 cells predicted from list of significantly downregulated genes in each treatment group (Adj. $p < 0.05$, $\log_2$ FC < -1.5). **(D-F)** Upregulated pathways in drug-treated MP46 cells predicted from list of significantly upregulated genes in each treatment group (Adj. $p < 0.05$, $\log_2$ FC > 1.5). **(G-I)** Downregulated pathways in drug-treated MP46 cells predicted from list of significantly downregulated genes in each treatment group (Adj. $p < 0.05$, $\log_2$ FC < -1.5). **(J-L)** Upregulated pathways in drug-treated MP38 cells predicted from list of significantly upregulated genes in each treatment group (Adj. $p < 0.05$, $\log_2$ FC > 1.5). **(M-O)** Downregulated pathways in drug-treated MP38 cells predicted from list of significantly downregulated genes in each treatment group (Adj. $p < 0.05$, $\log_2$ FC < -1.5).

OriginalData: (originaldata.tif) Uncropped raw western images of **(A)** SYN1 (red) and ACTB (green) and **(B)** TUBB3 (red) and ACTB (green). Cropped versions shown in Fig. 6D and Fig. 6F.

Supplementary Data 1: (SupplementaryData_1.xlsx) Full list of compounds in the TargetMol L1200 library (July 2022) and mean percent cell viability relative to DMSO control for each cell line following 72 h treatment with 1 μM of each compound in the primary screen. Shown in Fig. 1A-C.

Supplementary Data 2: (SupplementaryData_2.xlsx) Transcriptional Consensus Signatures (TCS) of HDAC inhibitors obtained from the iLINCS CMAP-L1000 dataset. Negative TCS scores indicate downregulated genes and positive scores indicate upregulation. Full dataset available at LINCS LDS-1611 and described in the following publication: PMC11601385. Used for figures Fig. 4G, Supplementary Fig. 4F, and Supplementary Fig. 5F.

Supplementary Data 3: (SupplementData_3.xlsx) List of genes consistently up- or downregulated by HDAC inhibitor treatments based on TCS data presented in Supplementary Data 2. Genes were selected if they were dysregulated in the same direction by all 8 HDAC treatments listed. "+" indicates a positive TCS score for each HDAC inhibitor for given gene, indicating upregulation, while "-" indicates a negative TCS score for each HDAC inhibitor for given gene, indicating downregulation. Used for figures Fig. 4G, Supplementary Fig. 4F, and Supplementary Fig. 5F.

Supplementary Data 4: (SupplementaryData_4.xlsx) Correlation matrix showing the pairwise Pearson Correlation (r) of Transcriptional Consensus Signatures (TCS) derived from the iLINCS L1000 dataset (December 2015 version) and independently treated GBM cell lines, as previously described in Nature Communications 2018 (PMID: 30552330). The raw data matrix was provided upon request from the publication authors. Used for Fig. 4H-I.
