# Supplementary material for: Identification of targetable epigenetic vulnerabilities for uveal melanoma
Source: Cell Death Dis. 2025 Dec 12;17(1):89. doi: 10.1038/s41419-025-08295-4 (PMC12830624; doi:10.1038/s41419-025-08295-4)
Supplement: Supplementary file 2 — Supplementary Table 1 [file 41419_2025_8295_MOESM2_ESM.docx]

| Name | MP41 IC_50_ (M) | MP38 IC_50_ (M) | MP46 IC_50_ (M) | Average IC_50_ (M) | Mechanism |
| --- | --- | --- | --- | --- | --- |
| Romidepsin | 2.81E-09 | 3.92E-09 | 5.36E-09 | **4.03E-09** | Class I HDAC inhibitor |
| Quisinostat 2HCl | 5.42E-09 | 6.75E-09 | 1.39E-08 | **8.67E-09** | HDAC inhibitor |
| Fimepinostat | 5.96E-09 | 1.76E-08 | 9.29E-09 | **1.09E-08** | HDAC and PI3K inhibitor |
| Quisinostat | 9.12E-09 | 1.16E-08 | 1.98E-08 | **1.35E-08** | HDAC inhibitor |
| Panobinostat | 1.43E-08 | 3.58E-08 | 2.77E-08 | **2.59E-08** | HDAC inhibitor |
| AZD5153 | 9.04E-08 | 8.87E-08 | 6.83E-08 | **8.25E-08** | BRD4 inhibitor |
| Alobresib | 1.04E-07 | 1.40E-07 | 7.12E-08 | **1.05E-07** | BET inhibitor |
| Mivebresib | 9.96E-08 | 1.88E-07 | 8.92E-08 | **1.25E-07** | BET inhibitor |
| CPI203 | 1.53E-07 | 1.60E-07 | 1.09E-07 | **1.41E-07** | BRD4 inhibitor |
| GSK1324726A | 1.54E-07 | 2.48E-07 | 1.05E-07 | **1.69E-07** | BRD2, BRD3, BRD4 inhibitor |
| BET Bromodomain Inhibitor | 3.76E-07 | 3.45E-07 | 2.48E-07 | **3.23E-07** | BET inhibitor |
| Velcade | 4.34E-09 | 1.49E-08 | 3.52E-09 | **7.58E-09** | Proteosome inhibitor |
| Podofilox | 6.49E-09 | 1.50E-08 | 6.56E-09 | **9.36E-09** | Topoisomerase II inhibitor |
| Cucurbitacin B | 4.84E-08 | 1.41E-08 | 5.11E-08 | **3.79E-08** | PI3K/AKT inhibitor |
| (S)-(+)-Camptothecin | 1.17E-07 | 6.76E-07 | 2.10E-07 | **3.34E-07** | Topoisomerase I inhibitor |
| Staurosporine | 2.71E-07 | 5.19E-08 | 6.86E-07 | **3.36E-07** | PKCα, PKCγ, PKCη inhibitor |
| Gemcitabine | 7.69E-08 | 1.23E-06 | 1.69E-07 | **4.93E-07** | DNA synthesis inhibitor |
| NSC228155 | 2.59E-06 | 3.22E-07 | 2.79E-06 | **1.90E-06** | EGFR activator |
| SKLB-23bb | 8.44E-08 | 1.74E-05 | 9.48E-08 | **5.87E-06** | HDAC6 inhibitor |
| ABBV-744 | 8.127 | 2834 | 0.0566 | **947.4** | BRD4 inhibitor |
| 666-15 | 5.89E-05 | 0.000585 | 0.00219 | **0.000946** | EGFR inhibitor |
| KW2449 | 3.89E-05 | 0.000458 | Unstable | **N/A** | FLT/STAT Kinase inhibitor |
| 4100-3815 | Unstable | 0.000246 | Unstable | **N/A** | Ataxin inhibitor |
| Y031-1770 | Unstable | Unstable | Unstable | **N/A** | Ataxin inhibitor |
