# Supplementary figures and images for: Identification of targetable epigenetic vulnerabilities for uveal melanoma
Source: Cell Death Dis. 2025 Dec 12;17(1):89. doi: 10.1038/s41419-025-08295-4 (PMC12830624; doi:10.1038/s41419-025-08295-4)

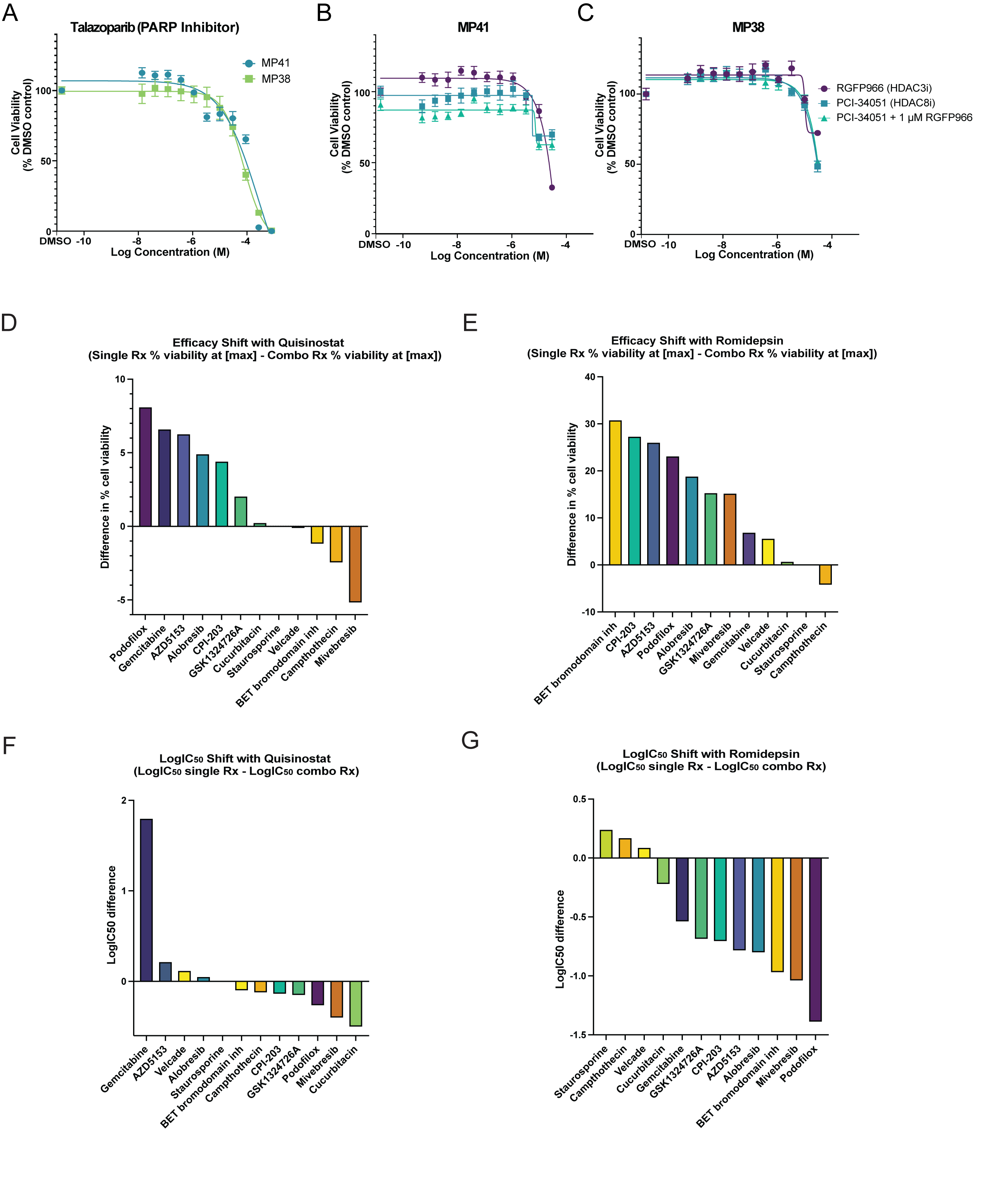

Supplement: Supplementary file 3 — Supplementary Figure 1 [file 41419_2025_8295_MOESM3_ESM.tif]

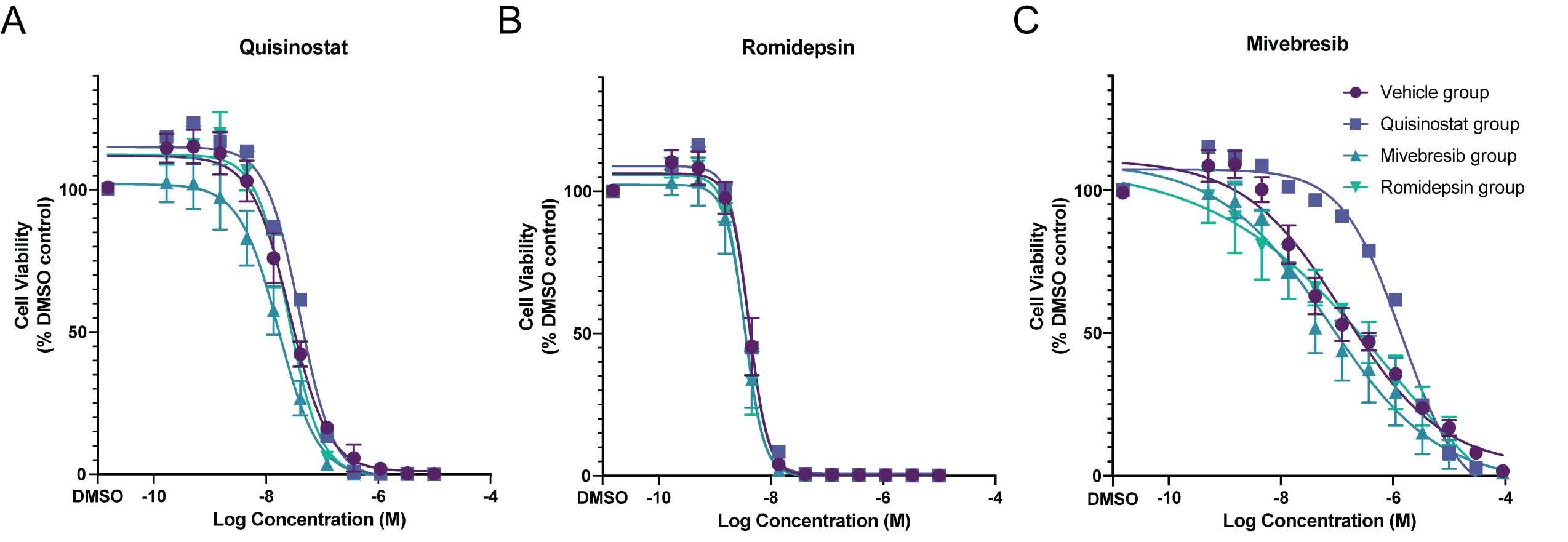

Supplement: Supplementary file 4 — Supplementary Figure 2 [file 41419_2025_8295_MOESM4_ESM.tif]

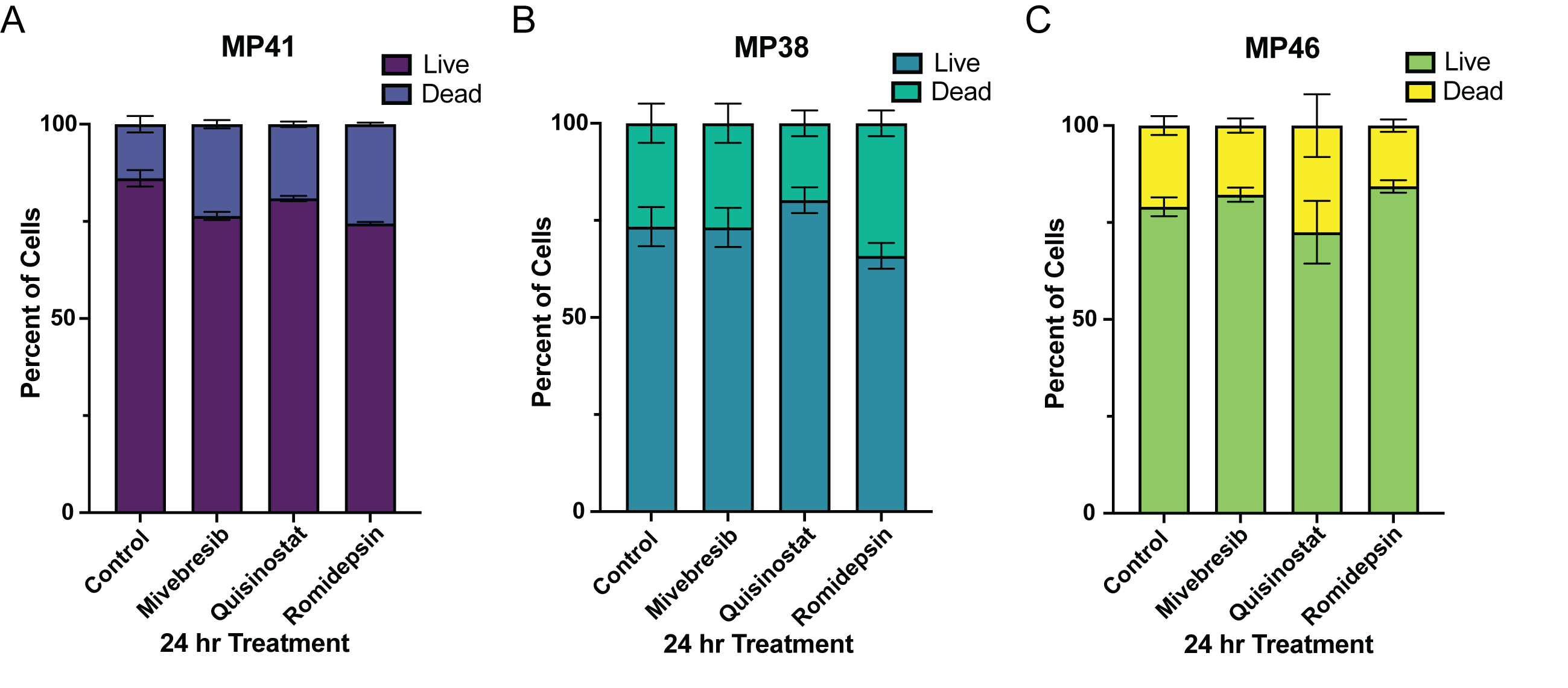

Supplement: Supplementary file 5 — Supplementary Figure 3 [file 41419_2025_8295_MOESM5_ESM.tif]

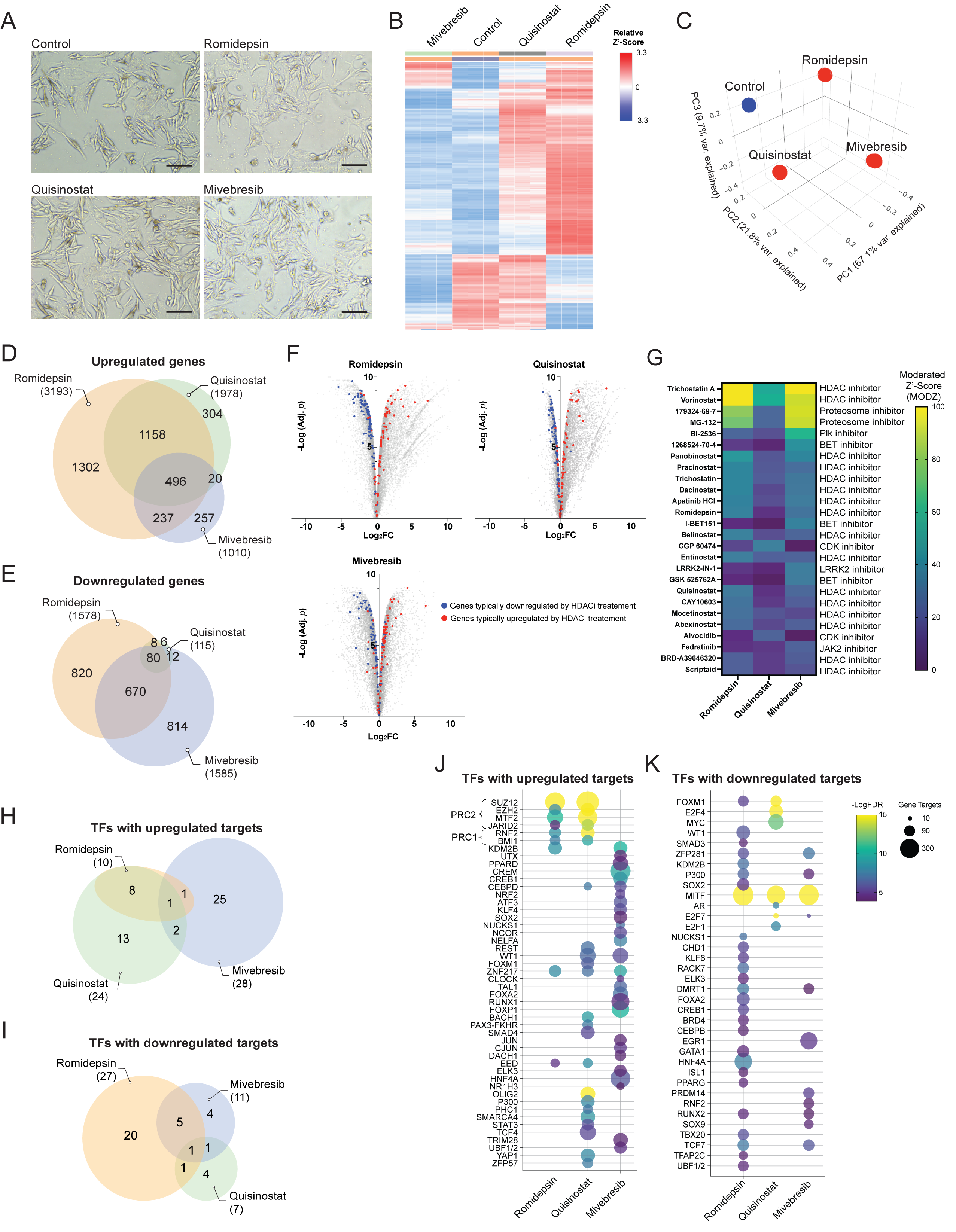

Supplement: Supplementary file 6 — Supplementary Figure 4 [file 41419_2025_8295_MOESM6_ESM.tif]

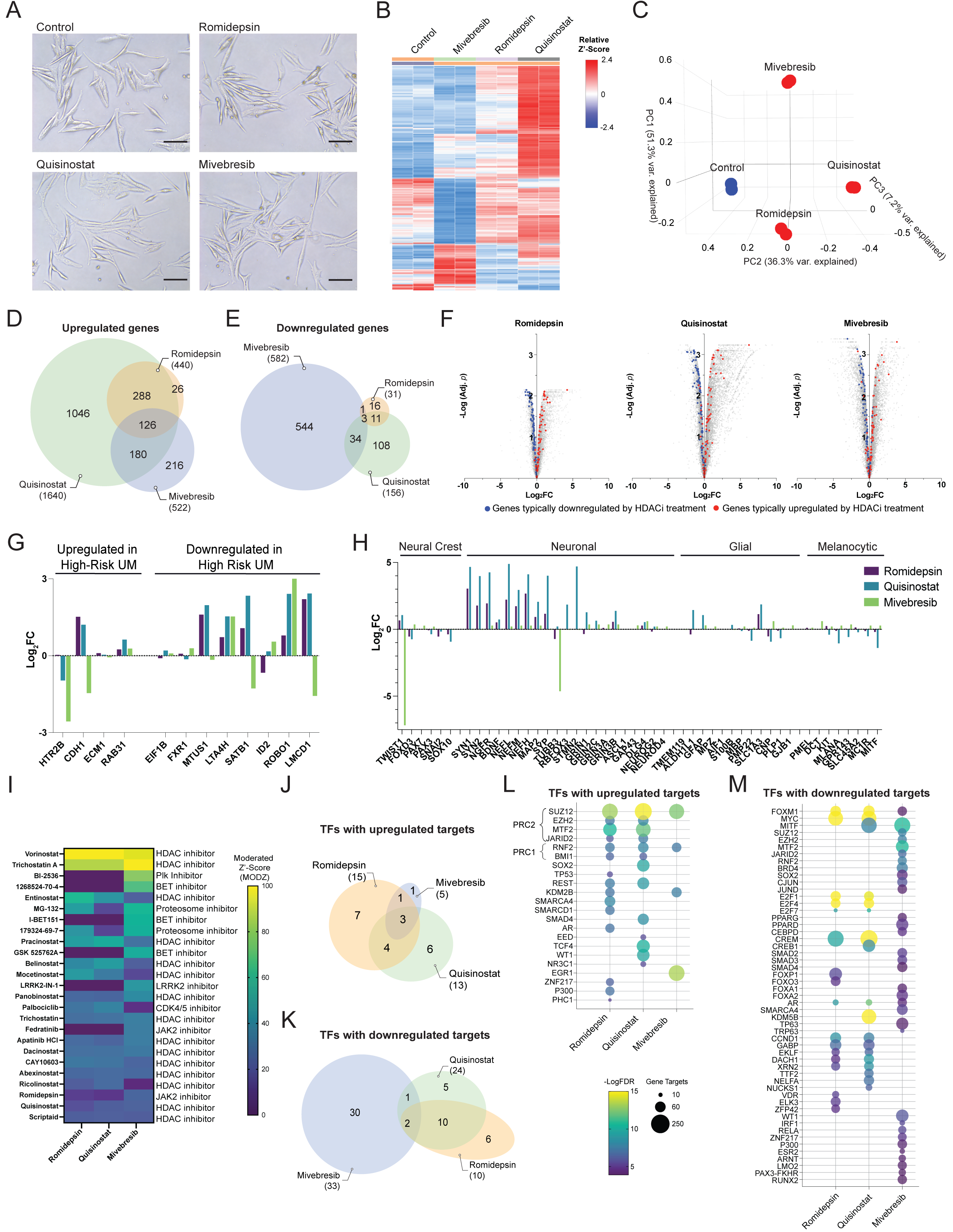

Supplement: Supplementary file 7 — Supplementary Figure 5 [file 41419_2025_8295_MOESM7_ESM.tif]

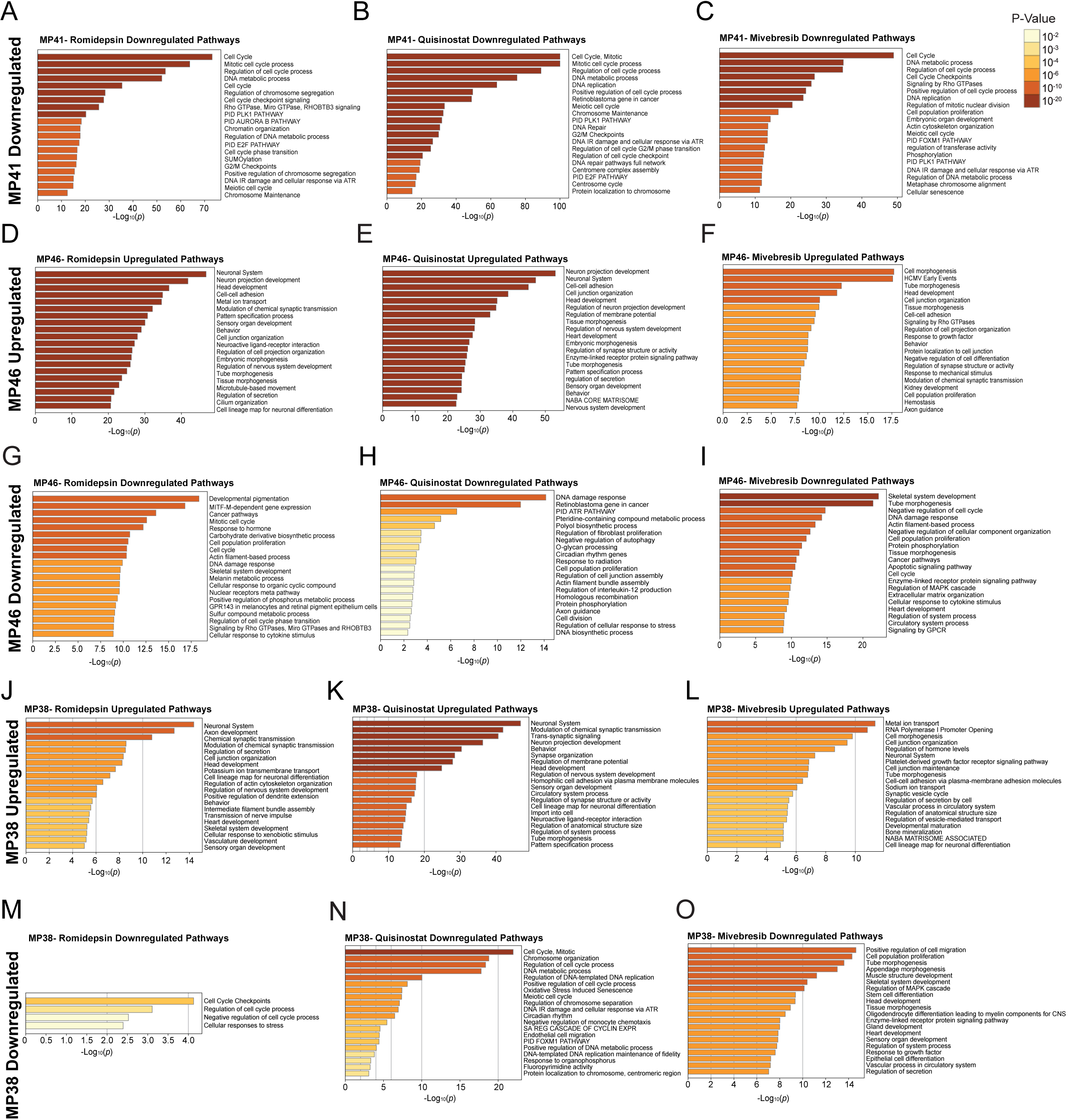

Supplement: Supplementary file 8 — Supplementary Figure 6 [file 41419_2025_8295_MOESM8_ESM.tif]

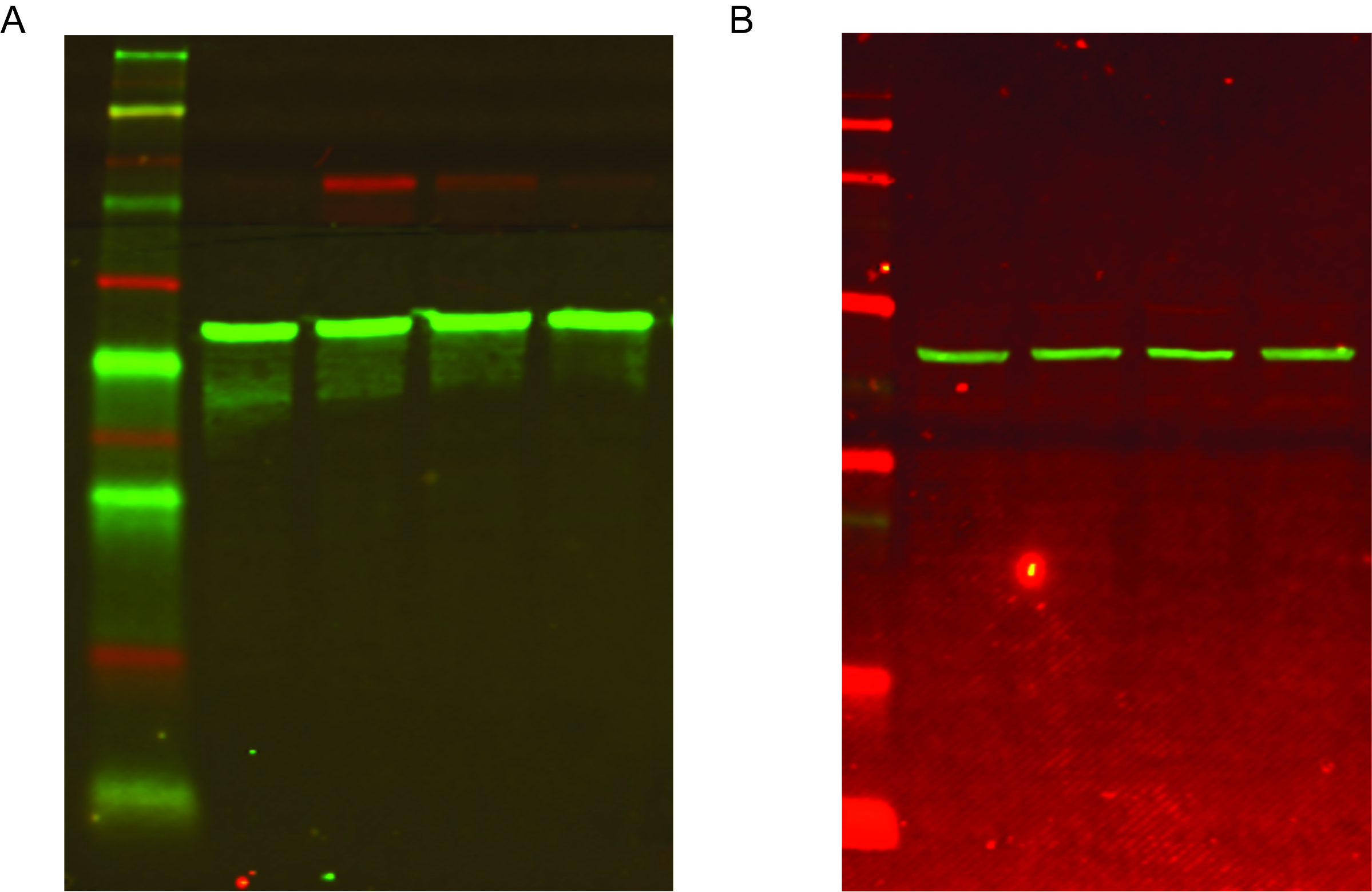

Supplement: Supplementary file 9 — OriginalData [file 41419_2025_8295_MOESM9_ESM.tif]
